# Time series analysis for psychological research: examining and forecasting change

**DOI:** 10.3389/fpsyg.2015.00727

**Published:** 2015-06-09

**Authors:** Andrew T. Jebb, Louis Tay, Wei Wang, Qiming Huang

**Affiliations:** ^1^Department of Psychological Sciences, Purdue UniversityWest Lafayette, IN, USA; ^2^Department of Psychology, University of Central FloridaOrlando, FL, USA; ^3^Department of Statistics, Purdue UniversityWest Lafayette, IN, USA

**Keywords:** time series analysis, longitudinal data analysis, forecasting, regression analysis, ARIMA

## Abstract

Psychological research has increasingly recognized the importance of integrating temporal dynamics into its theories, and innovations in longitudinal designs and analyses have allowed such theories to be formalized and tested. However, psychological researchers may be relatively unequipped to *analyze* such data, given its many characteristics and the general complexities involved in longitudinal modeling. The current paper introduces time series analysis to psychological research, an analytic domain that has been essential for understanding and predicting the behavior of variables across many diverse fields. First, the characteristics of time series data are discussed. Second, different time series modeling techniques are surveyed that can address various topics of interest to psychological researchers, including describing the pattern of change in a variable, modeling seasonal effects, assessing the immediate and long-term impact of a salient event, and forecasting future values. To illustrate these methods, an illustrative example based on online job search behavior is used throughout the paper, and a software tutorial in R for these analyses is provided in the Supplementary Materials.

Although time series analysis has been frequently used many disciplines, it has not been well-integrated within psychological research. In part, constraints in data collection have often limited longitudinal research to only a few time points. However, these practical limitations do not eliminate the *theoretical* need for understanding patterns of change over long periods of time or over many occasions. Psychological processes are inherently time-bound, and it can be argued that no theory is truly time-independent (Zaheer et al., 1999). Further, its prolific use in economics, engineering, and the natural sciences may perhaps be an indicator of its potential in our field, and recent technological growth has already initiated shifts in data collection that proliferate time series designs. For instance, online behaviors can now be quantified and tracked in real-time, leading to an accessible and rich source of time series data (see Stanton and Rogelberg, 2001). As a leading example, Ginsberg et al. (2009) developed methods of influenza tracking based on Google queries whose efficiency surpassed conventional systems, such as those provided by the Center for Disease Control and Prevention. Importantly, this work was based in prior research showing how search engine queries correlated with virological and mortality data over multiple years (Polgreen et al., 2008).

Furthermore, although experience sampling methods have been used for decades (Larson and Csikszentmihalyi, 1983), nascent technologies such as smartphones allow this technique to be increasingly feasible and less intrusive to respondents, resulting in a proliferation of time series data. As an example, Killingsworth and Gibert (2010) presented an iPhone (Apple Incorporated, Cupertino, California) application which tracks various behaviors, cognitions, and affect over time. At the time their study was published, their database contained almost a quarter of a million psychological measurements from individuals in 83 countries. Finally, due to the growing synthesis between psychology and neuroscience (e.g., affective neuroscience, social-cognitive neuroscience) the ability to analyze neuroimaging data, which is strongly linked to time series methods (e.g., Friston et al., 1995, 2000), is a powerful methodological asset. Due to these overarching trends, we expect that time series data will become increasingly prevalent and spur the development of more time-sensitive psychological theory. Mindful of the growing need to contribute to the methodological toolkit of psychological researchers, the present article introduces the use of *time series analysis* in order to describe and understand the dynamics of psychological change over time.

In contrast to these *current* trends, we conducted a survey of the existing psychological literature in order to quantify the extent to which time series methods have *already* been used in psychological science. Using the PsycINFO database, we searched the publication histories of 15 prominent journals in psychology^1^ for the term “time series” in the abstract, keywords, and subject terms. This search yielded a small sample of 36 empirical papers that utilized time series modeling. Further investigation revealed the presence of two general analytic goals: relating a time series to other substantive variables (17 papers) and examining the effects of a critical event or intervention (9 papers; the remaining papers consisted of other goals). Thus, this review not only demonstrates the relative scarcity of time series methods in psychological research, but also that scholars have primarily used *descriptive* or causal *explanatory* models for time series data analysis (Shmueli, 2010).

The prevalence of these types of models is typical of social science, but in fields where time series analysis is most commonly found (e.g., econometrics, finance, the atmospheric sciences), forecasting is often the primary goal because it bears on important practical decisions. As a result, the statistical time series literature is dominated by models that are aimed toward *prediction*, not explanation (Shmueli, 2010), and almost every book on applied time series analysis is exclusively devoted to forecasting methods (McCleary et al., 1980, p. 205). Although there are many well-written texts on time series modeling for economic and financial applications (e.g., Rothman, 1999; Mills and Markellos, 2008), there is a lack of formal introductions geared toward psychological issues (see West and Hepworth, 1991 for an exception). Thus, a psychologist looking to use these methodologies may find themselves with resources that focus on entirely different goals. The current paper attempts to amend this by providing an introduction to time series methodologies that is oriented toward issues within psychological research. This is accomplished by first introducing the basic characteristics of time series data: the four components of variation (trend, seasonality, cycles, and irregular variation), autocorrelation, and stationarity. Then, various time series regression models are explicated that can be used to achieve a wide range of goals, such as describing the process of change through time, estimating seasonal effects, and examining the effect of an intervention or critical event. Not to overlook the potential importance of forecasting for psychological research, the second half of the paper discusses methods for modeling autocorrelation and generating accurate predictions—viz., *autoregressive integrative moving average* (ARIMA) modeling. The final section briefly describes how regression techniques and ARIMA models can be combined in a *dynamic* regression model that can simultaneously explain and forecast a time series variable. Thus, the current paper seeks to provide an integrative resource for psychological researchers interested in analyzing time series data which, given the trends described above, are poised to become increasingly prevalent.

## The current illustrative application

**Figure 1:**
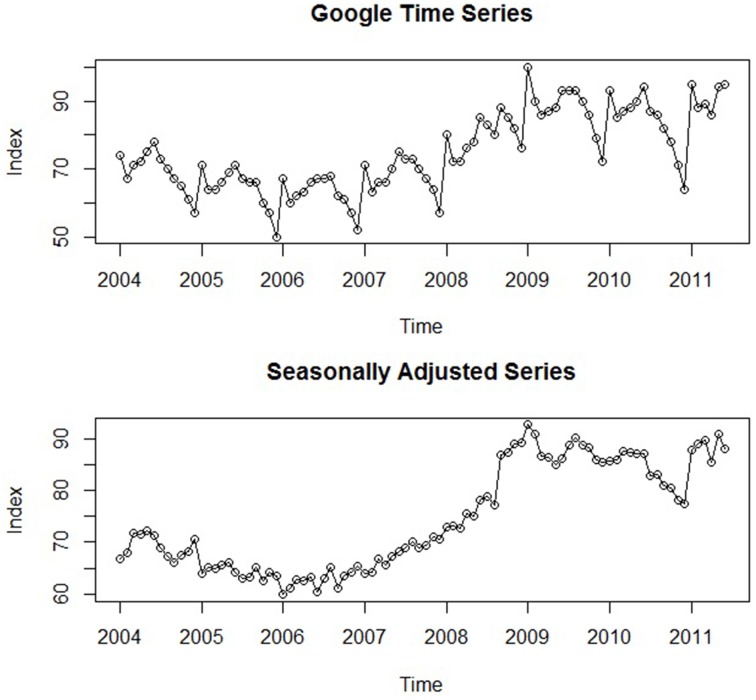
**A plot of the original Google job search time series and the series after seasonal adjustment**.

In order to better demonstrate how time series analysis can accomplish the goals of psychological research, a running practical example is presented throughout the current paper. For this particular illustration, we focused on online job search behaviors using data from Google Trends, which compiles the frequency of online searches on Google over time. We were particularly interested in the frequency of online job searches in the United States^2^ and the impact of the 2008 economic crisis on these rates. Our primary research hypothesis was that this critical event resulted in a sharp increase in the series that persisted over time. The monthly frequencies of these searches from January 2004 to June 2011 were recorded, constituting a data set of 90 total observations. Figure 1 displays a plot of this original time series that will be referenced throughout the current paper. Importantly, the values of the series do not represent the raw number of Google searches, but have been normalized (0–100) in order to yield a more tractable data set; each monthly value represents its percentage relative to the maximum observed value^3^.

## A note on software implementation

Conceptual expositions of new analytical methods can often be undermined by the practical issue of software implementation (Sharpe, 2013). To preempt this obstacle, for each analysis we provide accompanying R code in the Supplementary Material, along with an intuitive explanation of the meanings and rationale behind the various commands and arguments. On account of its versatility, the open-source statistical package R (R Development Core Team, 2011) remains the software platform of choice for performing time series analyses, and a number of introductory texts are oriented solely toward this program, such as *Introductory Time Series with R* (Cowpertwait and Metcalfe, 2009), *Time Series Analysis with Applications in R* (Cryer and Chan, 2008), and *Time Series Analysis and Its Applications with R Examples* (Shumway and Stoffer, 2006). In recent years, *R* has become increasingly recognized within the psychological sciences as well (Muenchen, 2013). We believe that psychological researchers with even a minimal amount of experience with R will find this tutorial both informative and accessible.

## An introduction to time series data

Before introducing how time series analyses can be used in psychological research, it is necessary to first explicate the features that characterize time series data. At its simplest, a time series is a set of time-ordered observations of a process where the intervals between observations remain constant (e.g., weeks, months, years, and minor deviations in the intervals are acceptable; McCleary et al., 1980, p. 21; Cowpertwait and Metcalfe, 2009). Time series data is often distinguished from other types of longitudinal data by the *number* and *source* of the observations; a univariate time series contains many observations originating from a single source (e.g., an individual, a price index), while other forms of longitudinal data often consist of several observations from many sources (e.g., a group of individuals). The length of time series can vary, but are generally at least 20 observations long, and many models require at least 50 observations for accurate estimation (McCleary et al., 1980, p. 20). More data is always preferable, but at the very least, a time series should be long enough to capture the phenomena of interest.

Due to its unique structure, a time series exhibits characteristics that are either absent or less prominent in the kinds of cross-sectional and longitudinal data typically collected in psychological research. In the next sections, we review these features that include *autocorrelation* and *stationarity*. However, we begin by delineating the types of patterns that may be present within a time series. That is, the variation or movement in a series can be partitioned into four parts: the *trend, seasonal, cyclical*, and *irregular* components (Persons, 1919).

### The four components of time series

#### Trend

Trend refers to any systematic change in the level of a series—i.e., its long-term direction (McCleary et al., 1980, p. 31; Hyndman and Athanasopoulos, 2014). Both the direction and slope (rate of change) of a trend may remain constant or change throughout the course of the series. Globally, the illustrative time series shown in Figure 1 exhibits a positive trend: The level of the series at the end is *systematically* higher than at its beginning. However, there are sections in this particular series that do not exhibit the same rate of increase. The beginning of the series displays a slight negative trend, and starting approximately at 2006, the series significantly rises until 2009, after which a small downward trend may even be present.

Because a trend in the data represents a significant source of variability, it must be accounted for when performing any time series analysis. That is, it must be either (a) modeled explicitly or (b) removed through mathematical transformations (i.e., *detrending*; McCleary et al., 1980, p. 32). The former approach is taken when the trend is theoretically interesting—either on its own or in relation to other variables. Conversely, removing the trend (through methods discussed later) is performed when this component is not pertinent to the goals of the analysis (e.g., strict forecasting). The decision of whether to model or remove systematic components like a trend represents an important aspect of time series analysis. The various characteristics of time series data are either of theoretical interest—in which case they should be modeled—or not, in which case they should be removed so that the aspects that *are* of interest can be more easily analyzed. Thus, it is incumbent upon the analyst to establish the goals of the analysis and determine which components of a time series are of interest and treat them accordingly. This topic will be revisited throughout the forthcoming sections.

#### Seasonality

Unlike the trend component, the seasonal component of a series is a repeating *pattern* of increase and decrease in the series that occurs consistently throughout its duration. More specifically, it can be defined as a cyclical or repeating pattern of movement within a period of 1 year or less that is attributed to “seasonal” factors—i.e., those related to an aspect of the calendar (e.g., the months or quarters of a year or the days of a week; Cowpertwait and Metcalfe, 2009, p. 6; Hyndman and Athanasopoulos, 2014). For instance, restaurant attendance may exhibit a *weekly* seasonal pattern such that the weekends routinely display the highest levels within the series across weeks (i.e., the time period), and the first several weekdays are consistently the lowest. Retail sales often display a *monthly* seasonal pattern, where each month across yearly periods consistently exhibits the same relative position to the others: viz., a spike in the series during the holiday months and a marked decrease in the following months. Importantly, the pattern represented by a seasonal effect remains constant and occurs over the same duration on each occasion (Hyndman and Athanasopoulos, 2014).

Although its underlying pattern remains fixed, the *magnitude* of a seasonal effect may vary across periods. Seasonal effects can also be embedded within overarching trends. Along with a marked trend, the series in Figure 1 exhibits noticeable seasonal fluctuations as well; at the beginning of each year (i.e., after the holiday months), online job searches spike and then fall significantly in February. After February, they continue to rise until about July or August, after which the series significantly drops for the remainder of the year, representing the effects of seasonal employment. Notice the consistency of both the form (i.e., pattern of increase and decrease) and magnitude of this seasonal effect. The fact that online job search behavior exhibits seasonal patterns supports the idea that this behavior (and this example in particular) is representative of job search behavior in general. In the United States, thousands of individuals engage in seasonal work which results in higher unemployment rates in the beginning of each year and in the later summer months (e.g., July and August; The United States Department of Labor, Bureau of Labor Statistics, 2014), manifesting in a similar seasonal pattern of job search behavior.

One may be interested in the presence of seasonal effects, but once identified, this source of variation is often removed from the time series through a procedure known as *seasonal adjustment* (Cowpertwait and Metcalfe, 2009, p. 21). This is in keeping with the aforementioned theme: Once a systematic component has been identified, it must either be modeled or removed. The popularity of seasonal adjustment is due to the characteristics of seasonal effects delineated above: Unlike other more dynamic components of a time series, seasonal patterns remain consistent across periods and are generally similar in magnitude (Hyndman and Athanasopoulos, 2014). Their effects may also obscure other important features of time series—e.g., a previously unnoticed trend or cycles described in the following section. Put simply, “seasonal adjustment is done to simplify data so that they may be more easily interpreted…without a significant loss of information” (Bell and Hillmer, 1984, p. 301). Unemployment rates are often seasonally adjusted to remove the fluctuations due to the effects of weather, harvests, and school schedules that remain more or less constant across years. In our data, the seasonal effects of job search behavior are not of direct theoretical interest relative to other features of the data, such as the underlying trend and the impact of the 2008 economic crisis. Thus, we may prefer to work with the simpler seasonally adjusted series. The lower panel of Figure 1 displays the original Google time series after seasonal adjustment, and the Supplementary Material contains a description of how to implement this procedure in R. It can be seen that the trend is made notably clearer after removing the seasonal effects. Despite the spike at the very end, the suspected downward trend in the later part of the series is much more evident. This insight will prove to be important when selecting an appropriate time series model in the upcoming sections.

#### Cycles

A cyclical component in a time series is conceptually similar to a seasonal component: It is a pattern of fluctuation (i.e., increase or decrease) that reoccurs across periods of time. However, unlike seasonal effects whose duration is *fixed* across occurrences and are associated with some aspect of the calendar (e.g., days, months), the patterns represented by cyclical effects are not of fixed duration (i.e., their length often varies from cycle to cycle) and are not attributable to any naturally-occurring time periods (Hyndman and Athanasopoulos, 2014). Put simply, cycles are any non-seasonal component that varies in a recognizable pattern (e.g., business cycles; Hyndman and Athanasopoulos, 2014). In contrast to seasonal effects, cycles generally occur over a period lasting longer than 2 years (although they may be shorter), and the magnitude of cyclical effects is generally more variable than that of seasonal effects (Hyndman and Athanasopoulos, 2014). Furthermore, just as the previous two components—trend and seasonality—can be present with or without the other, a cyclical component may be present with any combination of the other two. For instance, a trend with an intrinsic seasonal effect can be embedded within a greater cyclical pattern that occurs over a period of several years. Alternatively, a cyclical effect may be present without either of these two systematic components.

In the 7 years that constitute the time series of Figure 1, there do not appear to be any cyclical effects. This is expected, as there are no strong theoretical reasons to believe that online or job search behavior is significantly influenced by factors that consistently manifest across a period of over one year. We have significant *a priori* reasons to believe that causal factors related to seasonality exist (e.g., searching for work after seasonal employment), but the same does not hold true for long-term cycles, and the time series is sufficiently long enough to capture any potential cyclical behavior.

#### Irregular variation (randomness)

While the previous three components represented three *systematic* types of time series variability (i.e., *signal*; Hyndman and Athanasopoulos, 2014), the irregular component represents statistical noise and is analogous to the error terms included in various types of statistical models (e.g., the random component in generalized linear modeling). It constitutes any remaining variation in a time series after these three systematic components have been partitioned out. In time series parlance, when this component is completely random (i.e., not autocorrelated), it is referred to as *white noise*, which plays an important role in both the theory and practice of time series modeling. Time series are assumed to be in part driven by a white noise process (explicated in a future section), and white noise is vital for judging the adequacy of a time series model. After a model has been fit to the data, the residuals form a time series of their own, called the *residual error series*. If the statistical model has been successful in accounting for all the patterns in the data (e.g., systematic components such as trend and seasonality), the residual error series should be nothing more than unrelated white noise error terms with a mean of zero and some constant variance. In other words, the model should be successful in extracting all the signal present in the data with only randomness left over (Cowpertwait and Metcalfe, 2009, p. 68). This is analogous to evaluating the residuals of linear regression, which should be normally distributed around a mean of zero.

#### Time series decomposition

**Figure 2:**
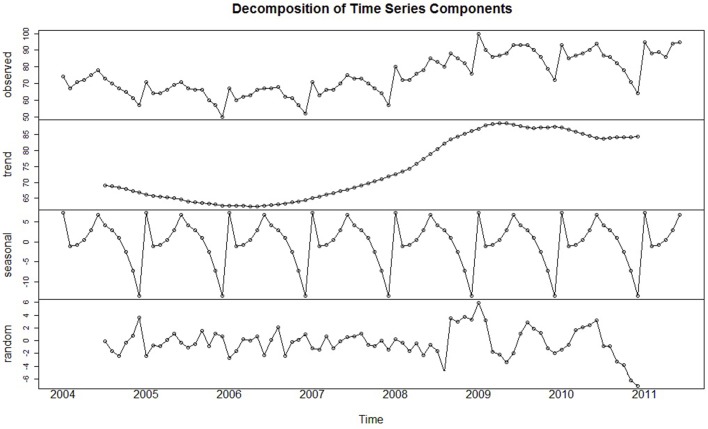
**The original time series decomposed into its trend, seasonal, and irregular (i.e., random) components**. Cyclical effects are not present within this series.

To visually examine a series in an exploratory fashion, time series are often formally partitioned into each of these components through a procedure referred to as *time series decomposition*. Figure 2 displays the original Google time series (top panel) decomposed into its constituent parts. This figure depicts what is referred to as *classical decomposition*, when a time series is conceived of comprising three components: a trend-cycle, seasonal, and random component. (Here, the trend and cycle are combined because the duration of each cycle is unknown; Hyndman and Athanasopoulos, 2014). The classic *additive decomposition model* (Cowpertwait and Metcalfe, 2009, p. 19) describes each value of the time series as the sum of these three components:

(1) $$y_{t}=T_{t}+S_{t}+E_{t}.$$

The additive decomposition model is most appropriate when the magnitude of the trend-cycle and seasonal components remain constant over the course of the series. However, when the magnitude of these components varies but still appears proportional over time (i.e., it changes by a multiplicative factor), the series may be better represented by the *multiplicative* decomposition model, where each observation is the *product* of the trend-cycle, seasonal, and random components:

(2) $$y_{t}=T_{t}\times S_{t}\times E_{t}.$$

In either decomposition model, each component is sequentially estimated and then removed until only the stochastic error component remains (the bottom panel of Figure 2). The primary purpose of time series decomposition is to provide the analyst with a better understanding of the underlying behavior and patterns of the time series which can be valuable in determining the goals of the analysis. Decomposition models can be used to generate forecasts by adding or multiplying future estimates of the seasonal and trend-cycle components (Hyndman and Athanasopoulos, 2014). However, such models are beyond the scope of this present paper, and the ARIMA forecasting models discussed later are generally superior^4^.

### Autocorrelation

In psychological research, the current state of a variable may partially depend on prior states. That is, many psychological variables exhibit *autocorrelation*: when a variable is correlated with itself across different time points (also referred to as *serial dependence*). Time series designs capture the effect of previous states and incorporate this potentially significant source of variance within their corresponding statistical models. Although the main features of many time series are its systematic components such as trend and seasonality, a large portion of time series methodology is aimed at explaining the autocorrelation in the data (Dettling, 2013, p. 2).

The importance of accounting for autocorrelation should not be overlooked; it is ubiquitous in social science phenomena (Kerlinger, 1973; Jones et al., 1977; Hartmann et al., 1980; Hays, 1981). In a review of 44 behavioral research studies with a total of 248 independent sets of repeated measures data, Busk and Marascuilo (1988) found that 80% of the calculated autocorrelations ranged from 0.1 to 0.49, and 40% exceeded 0.25. More specific to the psychological sciences, it has been proposed that state-related constructs at the individual-level, such as emotions and arousal, are often contingent on prior states (Wood and Brown, 1994). Using autocorrelation analysis, Fairbairn and Sayette (2013) found that alcohol use reduces emotional inertia, the extent to which prior affective states determine current emotions. Through this, they were able to marshal support for the theory of *alcohol myopia*, the intuitive but largely untested idea that alcohol allows a greater enjoyment of the present, and thus formally uncovered an affective motivation for alcohol use (and misuse). Further, using time series methods, Fuller et al. (2003) found that job stress in the present day was *negatively* related to the degree of stress in the preceding day. Accounting for autocorrelation can therefore reveal new information on the phenomenon of interest, as the Fuller et al. (2003) analysis led to the counterintuitive finding that lower stress was observed after prior levels had been high.

Statistically, *autocorrelation* simply represents the Pearson correlation for a variable with itself at a previous time period, referred to as the *lag* of the autocorrelation. For instance, the lag-1 autocorrelation of a time series is the correlation of each value with the immediately preceding observation; a lag-2 autocorrelation is the correlation with the value that occurred two observations before. The autocorrelation with respect to any lag can be computed (e.g., a lag-20 autocorrelation), and intuitively, the strength of the autocorrelation generally diminishes as the length of the lag increases (i.e., as the values become further removed in time).

**Figure 3:**
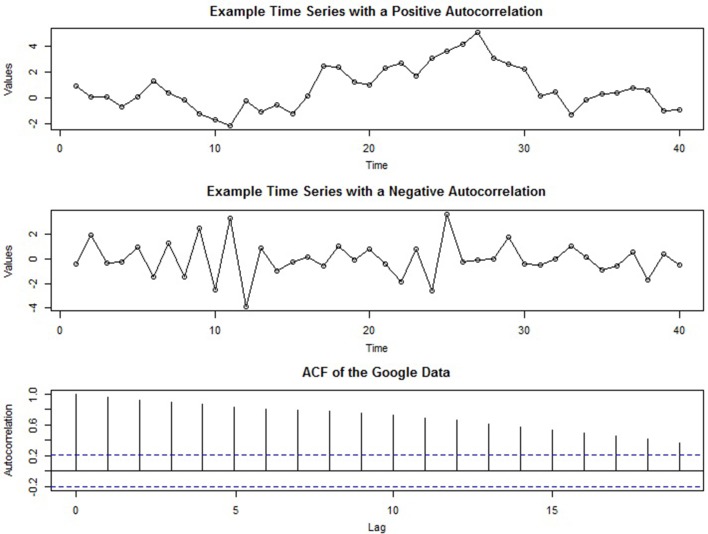
**Two example time series displaying exaggerated positive (top panel) and negative (center panel) autocorrelation**. The bottom panel depicts the ACF of the Google job search time series after seasonal adjustment.

Strong *positive* autocorrelation in a time series manifests graphically by “runs” of values that are either above or below the average value of the time series. Such time series are sometimes called “persistent” because when the series is above (or below) the mean value it tends to remain that way for several periods. Conversely, *negative* autocorrelation is characterized by the *absence* of runs—i.e., when positive values tend to follow negative values (and vice versa). Figure 3 contains two plots of time series intended to give the reader an intuitive understanding of the presence of autocorrelation: The series in the top panel exhibits positive autocorrelation, while the center panel illustrates negative autocorrelation. It is important to note that the autocorrelation in these series is not obscured by other components and that in real time series, visual analysis alone may not be sufficient to detect autocorrelation.

In time series analysis, the autocorrelation coefficient across many lags is called the *autocorrelation function* (ACF) and plays a significant role in model selection and evaluation (as discussed later). A plot of the ACF of the Google job search time series after seasonal adjustment is presented in the bottom panel of Figure 3. In an ACF plot, the y-axis displays the strength of the autocorrelation (ranging from positive to negative 1), and the x-axis represents the length of the lags: from lag-0 (which will always be 1) to much higher lags (here, lag-19). The dotted horizontal line indicates the *p* < 0.05 criterion for statistical significance.

### Stationarity

#### Definition and purpose

A complication with time series data is that its mean, variance, or autocorrelation structure can vary over time. A time series is said to be *stationary* when these properties remain constant (Cryer and Chan, 2008, p. 16). Thus, there are many ways in which a series can be non-stationary (e.g., an increasing variance over time), but it can only be stationary in one-way (viz., when all of these features do not change).

Stationarity is a pivotal concept in time series analysis because descriptive statistics of a series (e.g., its mean and variance) are only accurate population estimates if they remain constant throughout the series (Cowpertwait and Metcalfe, 2009, pp. 31–32). With a stationary series, it will not matter when the variable is observed: “The properties of one section of the data are much like those of any other” (Chatfield, 2004, p. 13). As a result, a stationary series is easy to predict: Its future values will be similar to those in the past (Nua, 2014). As a result, stationarity is *the most important assumption* when making predictions based on past observations (Cryer and Chan, 2008, p. 16), and many times series models assume the series already is or can be transformed to stationarity (e.g., the broad class of ARIMA models discussed later).

**Figure 4:**
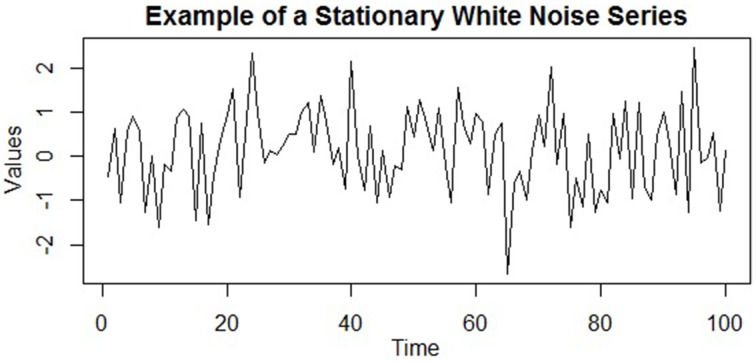
**An example of a stationary time series (specifically, a series of uncorrelated white noise terms)**. The mean, variance, and autocorrelation are all constant over time, and the series displays no systematic patterns, such as trends or cycles.

In general, a stationary time series will have no predictable patterns in the long-term; plots will show the series to be roughly horizontal with some constant variance (Hyndman and Athanasopoulos, 2014). A stationary time series is illustrated in Figure 4, which is a stationary white noise series (i.e., a series of uncorrelated terms). The series hovers around the same general region (i.e., its mean) with a consistent variance around this value. Despite the observations having a constant mean, variance, and autocorrelation, notice how such a process can generate outliers (e.g., the low extreme value after t = 60), as well as runs of values that are both above or below the mean. Thus, stationarity does not preclude these *temporary* and fluctuating behaviors of the series, although any *systematic* patterns would.

However, many time series in real life are dominated by trends and seasonal effects that preclude stationarity. A series with a trend cannot be stationary because, by definition, a trend is when the mean level of the series changes over time. Seasonal effects also preclude stationarity, as they are reoccurring patterns of change in the mean of the series within a fixed time period (e.g., a year). Thus, trend and seasonality are the two time series components that must be addressed in order to achieve stationarity.

#### Transforming a series to stationarity

When a time series is not stationary, it can be made so after accounting for these systematic components within the model or through mathematical transformations. The procedure of seasonal adjustment described above is a method that removes the systematic seasonal effects on the mean level of the series.

The most important method of stationarizing the mean of a series is through a process called *differencing*, which can be used to remove any trend in the series which is not of interest. In the simplest case of a linear trend, the slope (i.e., the change from one period to the next) remains relatively constant over time. In such a case, *the difference* between each time period and its preceding one (referred to as the first *differences*) are approximately equal. Thus, one can effectively “detrend” the series by transforming the original series into *a series* of first differences (Meko, 2013; Hyndman and Athanasopoulos, 2014). The underlying logic is that forecasting *the change* in a series from one period to the next is just as useful in practice as predicting the original series values.

However, when the time series exhibits a trend that itself changes (i.e., a non-constant slope), then even transforming a series into a series of its first differences may not render it completely stationary. This is because when the slope itself is changing (e.g., an exponential trend), the difference between periods will be unequal. In such cases, taking the first differences of the *already differenced series* (referred to as the second *differences*) will often stationarize the series. This is because each successive differencing has the effect of reducing the overall variance of the series (Anderson, 1976), as deviations from the mean level are increasingly reduced through this subtractive process. The second differences (i.e., the first differences of the already differenced series) will therefore further stabilize the mean. There are general guidelines on how many orders of differencing are necessary to stationarize a series. For instance, the first or second differences will nearly always stationarize the mean, and in practice it is almost never necessary to go beyond second differencing (Cryer and Chan, 2008; Hyndman and Athanasopoulos, 2014). However, for series that exhibit higher-degree polynomial trends, the order of differencing required to stationarize the series is typically equal to that degree (e.g., two orders of differencing for an approximately quadratic trend, three orders for a cubic trend; Cowpertwait and Metcalfe, 2009, p. 93).

A common mistake in time series modeling to “overdifference” the series, when more orders of differencing than are required to achieve stationarity are performed. This can complicate the process of building an adequate and parsimonious model (see McCleary et al., 1980, p. 97). Fortunately, overdifferencing is relatively easy to identify; differencing a series with a trend will have the effect of *reducing* the variance of the series, but an unnecessary degree of differencing will *increase* its variance (Anderson, 1976). Thus, the optimal order of differencing is that which results in the lowest variance of the series.

If the variance of a times series is not constant over time, a common method of making the variance stationary is through a logarithmic transformation of the series (Cowpertwait and Metcalfe, 2009, pp. 109–112; Hyndman and Athanasopoulos, 2014). Taking the logarithm has the practical effect of reducing each value at an exponential rate. That is, the larger the value, the more its value is reduced. Thus, this transformation stabilizes the differences across values (i.e., its variance) which is also why it is frequently used to mitigate the effect of outliers (e.g., Aguinis et al., 2013). It is important to remember that if one applies a transformation, any forecasts generated by the selected model will be in these transformed units. However, once the model is fitted and the parameters estimated, one can reverse these transformations to obtain forecasts in its original metric.

Finally, there are also formal statistical tests for stationarity, termed *unit root* tests. A very popular procedure is the *augmented Dickey–Fuller* test (ADF; Said and Dickey, 1984) which tests the null hypothesis that the series is non-stationary. Thus, rejection of the null provides evidence for a stationary series. Table 1 below contains information regarding the ADF test, as well as descriptions of other various statistical tests frequently used in time series analysis that will be discussed in the remainder of the paper. By using the ADF test in conjunction with the transformations described above (or the modeling procedures delineated below), an analyst can ensure that a series conforms to stationarity.

**Table 1:** **Common tests in time series analysis**.

**Test name**	**Null hypothesis**	**Primary use in modeling**
Augmented Dickey–Fuller (ADF)	The series is non-stationary; rejection implies a stationary series.	A series must be stationary before any AR or MA terms are added to account for its autocorrelation. The ADF test identifies if a series needs to be made stationary through differencing, or, after an order of differencing has been applied, if the series has indeed become stationary.
Durbin–Watson	The residuals from a regression model do not have a lag-1 autocorrelation; rejection implies lag-1 autocorrelated errors.	A Durbin–Watson test can assess if the residuals of a regression model are autocorrelated. When this is the case, including ARIMA terms or using generalized least squares estimation can account for this autocorrelation.
Ljung–Box	The errors are uncorrelated; rejection implies correlated errors.	After fitting an ARIMA or dynamic regression model to a series, the Ljung–Box test identifies if the model has been successful in extracting all the autocorrelation.

*There are other tests for stationarity, such as the Phillips–Perron and Kwiatkowski–Phillips–Schmidt–Shin (KPSS) tests which can sometimes yield contrary results. The ADF test was chosen as the focus of this paper due to its popularity and reliability. For information regarding the others, see Cowpertwait and Metcalfe (2009, pp. 214–215) and Hyndman and Athanasopoulos (2014)*.

## Time series modeling: regression methods

**Figure 5:**
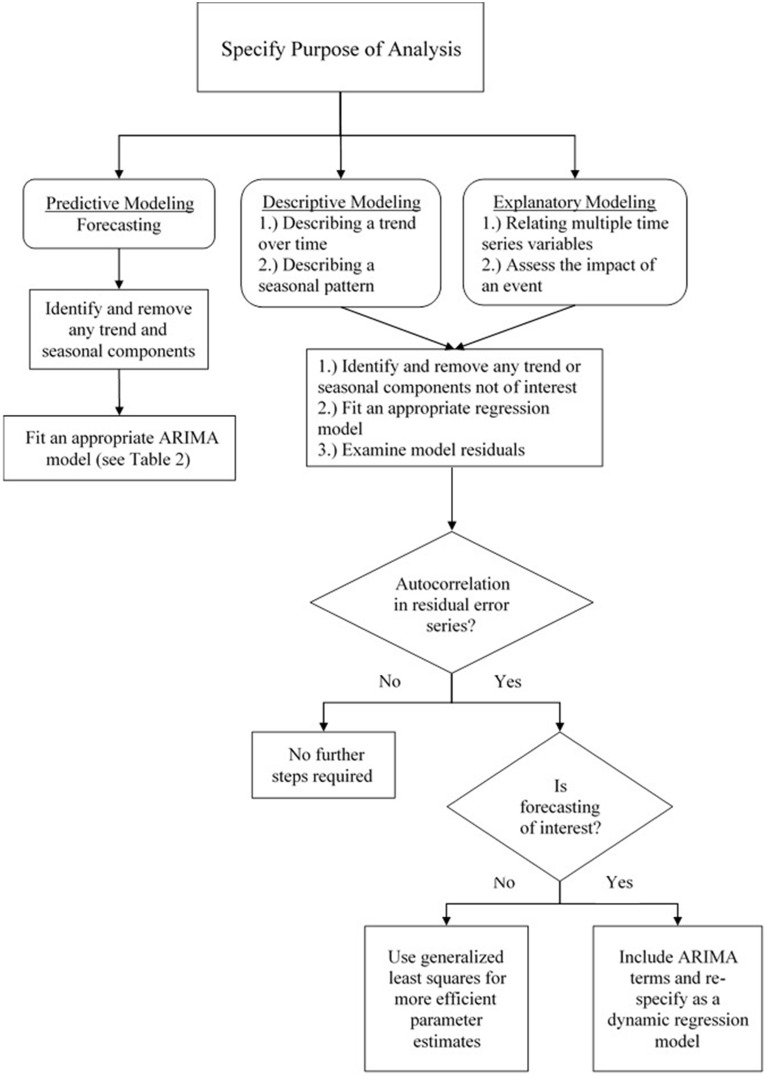
**A flowchart depicting various time series modeling approaches and how they are suited to address various goals in psychological research**.

The statistical time series literature is dominated by methodologies aimed at forecasting the behavior of a time series (Shmueli, 2010). Yet, as the survey in the introduction illustrated, psychological researchers are primarily interested in other applications, such as describing and accounting for an underlying trend, linking explanatory variables to the criterion of interest, and assessing the impact of critical events. Thus, psychological researchers will primarily use *descriptive* or *explanatory* models, as opposed to predictive models aimed solely at generating accurate forecasts. In time series analysis, each of the aforementioned goals can be accomplished through the use of regression methods in a manner very similar to the analysis of cross-sectional data. After having explicated the basic properties of time series data, we now discuss these specific modeling approaches that are able fulfill these purposes. The next four sections begin by first providing an overview of each type of regression model, how psychological research stands to gain from the use of these methods, and their corresponding statistical models. We include mathematical treatments, but also provide conceptual explanations so that they may be understood in an accessible and intuitive manner. Additionally, Figure 5 presents a flowchart depicting different time series models and which approaches are best for addressing the various goals of psychological research. As the current paper continues, the reader will come to understand the meaning and structure of these models and their relation to substantive research questions.

It is important to keep in mind that time series often exhibit strong autocorrelation which often manifests in correlated residuals after a regression model has been fit. This violates the standard assumption of independent (i.e., uncorrelated) errors. In the section that follows these regression approaches, we describe how the remaining autocorrelation can be included in the model by building a *dynamic* regression model that includes ARIMA terms^5^. That is, a regression model can be first fit to the data for explanatory or descriptive modeling, and ARIMA terms can be fit to the residuals in order to account for any remaining autocorrelation and improve forecasts (Hyndman and Athanasopoulos, 2014). However, we begin by introducing regression methods separate from ARIMA modeling, temporarily setting aside the issue of autocorrelation. This is done in order to better focus on the implementation of these models, but also because violating this assumption has minimal effects on the substance of the analysis: The parameter estimates remain unbiased and can still be used for prediction. Its forecasts will not be “wrong,” but *inefficient*—i.e., ignoring the information represented by the autocorrelation that could be used to obtain better predictions (Hyndman and Athanasopoulos, 2014). Additionally, *generalized least squares* estimation (as opposed to ordinary least squares) takes into account the effects of autocorrelation which otherwise lead to underestimated standard errors (Cowpertwait and Metcalfe, 2009, p. 98). This estimation procedure was used for each of the regression models below. For further information on regression methods for time series, the reader is directed to Hyndman and Athanasopoulos (2014, chaps. 4, 5) and McCleary et al. (1980), which are very accessible introductions to the topic, as well as Cowpertwait and Metcalfe (2009, chap. 5) and Cryer and Chan (2008, chaps. 3, 11) for more mathematically-oriented treatments.

### Modeling trends through regression

Modeling an observed trend in a time series through regression is appropriate when the trend is *deterministic*—i.e., the trend is due to the constant, deterministic effects of a few causal forces (McCleary et al., 1980, p. 34). As a result, a deterministic trend is generally stable across time. Expecting any trend to continue *indefinitely* is often unrealistic, but for a deterministic trend, linear extrapolation can provide accurate forecasts for several periods ahead, as forecasting generally assumes that trends will continue and change relatively slowly (Cowpertwait and Metcalfe, 2009, p. 6). Thus, when the trend is deterministic, it is desirable to use a regression model that includes the hypothesized causal factors as predictors (Cowpertwait and Metcalfe, 2009, p. 91; McCleary et al., 1980, p. 34).

Deterministic trends stand in contrast to *stochastic* trends, those that arise simply from the random movement of the variable over time (long runs of similar values due to autocorrelation; Cowpertwait and Metcalfe, 2009, p. 91). As a result, stochastic trends often exhibit frequent and inexplicable changes in both slope and direction. When the trend is deemed to be stochastic, it is often removed through differencing. There are also methods for forecasting using stochastic trends (e.g., *random walk* and *exponential smoothing* models) discussed in Cowpertwait and Metcalfe (2009, chaps. 3, 4) and Hyndman and Athanasopoulos (2014, chap. 7). However, the reader should be aware that these are predictive models only, as there is nothing about a stochastic trend that can be explained through external, theoretically interesting factors (i.e., it is a trend attributable to randomness). Therefore, attempting to model it deterministically as a function of time or other substantive variables via regression can lead to spurious relationships (Kuljanin et al., 2011) and inaccurate forecasts, as the trend is unlikely to remain stable over time.

Returning to the example Google time series of Figure 1, the evident trend in the seasonally adjusted series might appear to be stochastic: It is not constant but changes at several points within the series. However, we have strong theoretical reasons for modeling it deterministically, as the 2008 economic crisis is one causal factor that likely had a profound impact on the series. Thus, this theoretical rationale implies that the otherwise inexplicable changes in its trend are due to systematic forces that can be appropriately modeled within an explanatory approach (i.e., as a deterministic function of predictors).

#### The linear regression model

As noted in the literature review, psychological researchers are often directly interested in describing an underlying trend. For example, Fuller et al. (2003) examined the strain of university employees using a time series design. They found that each self-report item displayed the same deterministic trend: Globally, strain increased over time even though the perceived severity of the stressful events did not increase. Levels of strain also decreased at spring break and after finals week, during which mood and job satisfaction also exhibited rising levels. This finding cohered with prior theory on the accumulating nature of stress and the importance of regular strain relief (e.g., Bolger et al., 1989; Carayon, 1995). Furthermore, Wagner et al. (1988) examined the trend in employee productivity after the implementation of an incentive-based wage system. In addition to discovering an immediate increase in productivity, it was found that productivity increased over time as well (i.e., a continuing deterministic trend). This trend gradually diminished over time, but was still present at the end of the study period—nearly 6 years after the intervention first occurred.

By visually examining a time series, an analyst can describe how a trend changes as function of time. However, one can formally assess the behavior of a trend by regressing the series on a variable that represents time (e.g., 1–50 for 50 equally-spaced observations). In the simplest case, the trend can be modeled as a linear function of time, which is conceptually identical to a regression model for cross-sectional data using a single predictor:

(3) $$y_{t}=b_{0}+b_{1}t+\varepsilon_{t},$$

where the coefficient *b*_1_ estimates the amount of change in the time series associated with a one-unit increase in time, *t* is the time variable, and ε*_t_* is random error. The constant, *b*_0_, estimates the level of the series when *t* = 0.

**Figure 6:**
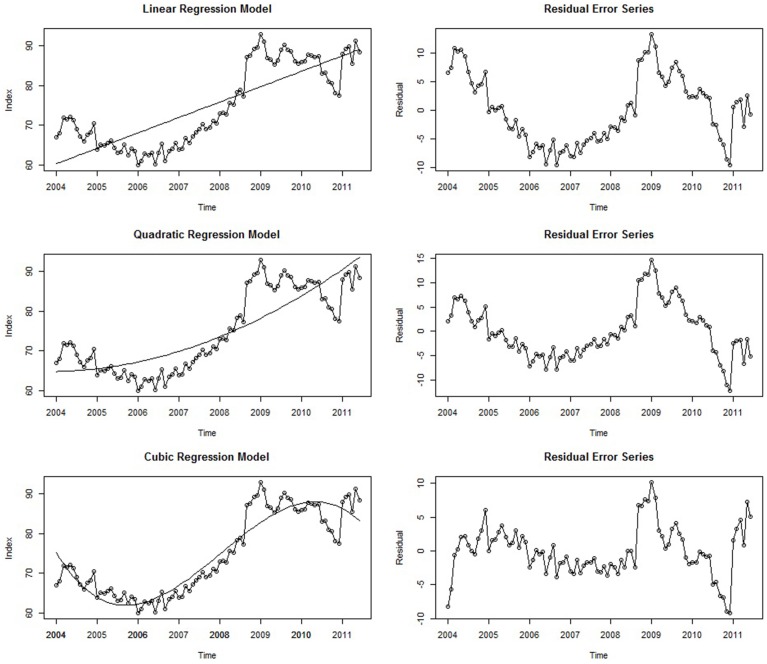
**Three different regression models with time as the regressor and their associated residual error series**.

If a deterministic trend is fully accounted for by a linear regression model, the residual error series (i.e., the collection of residuals which themselves form a time series) will not contain any remaining trend component; that is, this non-stationary behavior of the series will have been accounted for Cowpertwait and Metcalfe (2009), (p. 121). Returning to our empirical example, the linear regression model displayed in Equation (3) was fit to the seasonally adjusted Google job search data. This is displayed in the top left panel of Figure 6. The regression line of best-fit is superimposed, and the residual error series is shown in the panel directly to the right. Here, time is a significant predictor (*b*_1_ = 0.32, *p* < 0.001), and the model accounts for 67% of the seasonally-adjusted series variance (*R*^2^ = 0.67, *p* < 0.001). However, the residual error series displays a notable amount of remaining trend that has been left unaccounted for; the first half of the error series has a striking downward trend that begins to rise at around 2007. This is because the regression line is constrained to linearity and therefore *systematically* underestimates and overestimates the values of the series when the trend exhibits runs of high and low values, respectively. Importantly, the forecasts from the simple linear model will most likely be very poor as well. Although there is a spike at the end of the series, the linear model predicts that values further ahead in time will be even higher. By contrast, we actually expect these values to decrease, similar to how there was a decreasing trend in 2008 right after the first spike. Thus, despite accounting for a considerable amount of variance and serving as a general *approximation* of the series trend, the linear model is insufficient in several systematic ways, manifesting in inaccurate forecasts and a significant remaining trend in the residual error series. A method for improving this model is to add in a higher-order polynomial term; modeling the trend as quadratic, cubic, or an even higher-order function may lead to a better-fitting model, but the analyst must be vigilant of overfitting the series—i.e., including so many parameters that the statistical noise becomes modeled. Thus, striking a balance between parsimony and explanatory capability should always be a consideration when modeling time series (and statistical modeling in general). Although a simple linear regression on time is often adequate to approximate a trend (Cowpertwait and Metcalfe, 2009, p. 5), in this particular instance a higher-order term may provide a better fit to the complex deterministic trend seen within this series.

#### Polynomial regression models

When describing the trend in the Google data earlier, it was noted that the series began to display a rising trend approximately a third of the way into the series, implying that a *quadratic* regression model (i.e., a single bend) may yield a good fit to the data. Furthermore, our initial hypothesis was that job search behavior proceeded at a generally constant rate and then spiked once the economic crisis began—also implying a quadratic trend. In some time series, the trend over time will be non-linear, and the predictor terms can be specified to reflect such higher-order terms (quadratic, cubic, etc.). Just like when modeling cross-sectional data, non-linear terms can be incorporated into the statistical model by squaring the predictor (here, time)^6^:

(4) $$y_{t}=b_{0}+b_{1}t+b_{2}t^{2}+\varepsilon_{t}.$$

The center panels in Figure 6 show the quadratic model and its residual error series. In line with the initial hypothesis, both the quadratic term (*b*_2_ = 0.003, *p* < 0.001) and linear term (*b*_1_ = 0.32, *p* < 0.001) were statistically significant. Thus, modeling the trend as a quadratic function of time explained an additional 4% of the series variance relative to the more parsimonious linear model (*R*^2^ = 0.71, *p* < 0.001). However, examination of this series and its residuals shows that it is not as different from the linear model than was expected; although the first half of the residual error series has a more stable mean level, there are still noticeable trends in the first half of the residual error series, and the forecasts implied by this model are even higher than those of the linear model. Therefore, a *cubic* trend may provide an even better fit, as there are two apparent bends in the series:

(5) $$y_{t}=b_{0}+b_{1}t+b_{2}t^{2}+b_{3}t^{3}+\varepsilon_{t}.$$

After fitting this model to the Google data, 87% of the series variance is accounted for (*R*^2^ = 0.87 *p* < 0.001), and all three coefficients are statistically significant: *b*_1_ = 0.69, *p* < 0.001, *b*_2_ = 0.003, *p* = 0.05, and *b*_3_ = −0.0003, *p* < 0.001. Furthermore, the forecasts implied by the model are much more realistic. Ultimately, it is unlikely that this model will provide accurate forecasts many periods into the future (as is often the case for regression models; Cowpertwait and Metcalfe, 2009, p. 6; Hyndman and Athanasopoulos, 2014). It is more likely that either (a) a negative trend will return the series back to more moderate levels or (b) the series will simply continue at a generally high level. Furthermore, relative to the linear model, the residual error series of this model appears much closer to stationarity (e.g., Figure 4), as the initial downward trend of the time series is captured. Therefore, modeling the series as a cubic function of time is the most successful in terms of accounting for the trend, and adding an even higher-order polynomial term has little remaining variance to explain (<15%) and would likely lead to an overfitted model. Thus, relative to the two previous models, the cubic model strikes a balance between relative parsimony and descriptive capability. However, any forecasts from this model could be improved upon by removing the remaining trend and including other terms that account for any autocorrelation in the data, topics discussed in an upcoming section on ARIMA modeling.

### Interrupted time series analysis

#### Overview

Although we are interested in describing the underlying trend within the Google time series as a function of time, we are also interested in the effect of a critical event, represented by the following question: “Did the 2008 economic crisis result in elevated rates job search behaviors?” In psychological science, many research questions center on the impact of an event, whether it be a relationship change, job transition, or major stressor or uplift (Kanner et al., 1981; Dalal et al., 2014). In the survey of how time series analysis had been previously used in psychological research, examining the impact of an event was one of its most common uses. In time series methodology, questions regarding the impact of events can be analyzed through *interrupted time series analysis* (or *intervention analysis*; Glass et al., 1975), in which the time series observations are “interrupted” by an intervention, treatment, or incident occurring at a known point in time (Cook and Campbell, 1979).

In both academic and applied settings, psychological researchers are often constrained to correlational, cross-sectional data. As a result, researchers rarely have the ability to implement control groups within their study designs and are less capable of drawing conclusions regarding causality. In the majority of cases, it is the *theory* itself that provides the rationale for drawing causal inferences (Shmueli, 2010, p. 290). In contrast, an interrupted time series is the strongest quasi-experimental design to evaluate the longitudinal impact of an event (Wagner et al., 2002, p. 299). In a review of previous research on the efficacy of interventions, Beer and Walton (1987) stated, “much of the research overlooks time and is not sufficiently longitudinal. By assessing the events and their impact at only one nearly contemporaneous moment, the research cannot discuss how permanent the changes are” (p. 343). Interrupted time series analysis ameliorates this problem by taking multiple measurements both before and after the event, thereby allowing the analyst to examine the pre- and post-event trend.

Collecting data at multiple time points also offers advantages relative to cross-sectional comparisons based on pre- and post-event means. A longitudinal interrupted time series design allows the analyst to control for the trend *prior* to the event, which may turn out to be the cause of any alleged intervention effect. For instance, in the field of industrial/organizational psychology, Pearce et al. (1985) found a positive trend in four measures of organizational performance over the course of the 4 years under study. However, after incorporating the effects of the pre-event trend in the analysis, neither the implementation of the policy nor the first year of merit-based rewards yielded any additional effects. That is, the post-event trends were almost totally attributable to the pre-event behavior of the series. Thus, a time series design and analysis yielded an entirely different and more parsimonious conclusion that might have otherwise been drawn. In contrast, Wagner et al. (1988) was able to show that that for non-managerial employees, an incentive-based wage system substantially increased employee productivity in both its baseline level and post-intervention slope (the baseline level jumped over 100%). Thus, interrupted time series analysis is an ideal method for examining the impacts of such events and can be generalized to other criteria of interest.

#### Modeling an interrupted time series

Statistical modeling of an interrupted time series can be accomplished through *segmented regression analysis* (Wagner et al., 2002, p. 300). Here, the time series is partitioned into two parts: the pre- and post-event segments whose levels (intercepts) and trends (slopes) are both estimated. A change in these parameters represents an effect of the event: A significant change in the level of the series indicates an immediate change, and a change in trend reflects a more gradual change in the outcome (and of course, both are possible; Wagner et al., 2002, p. 300). The formal model reflects these four parameters of interest:

(6) $$y_{t}=b_{0}+b_{1}\times t+b_{2}\times event_{t}+b_{3}\times t\;after\;event+\;\varepsilon_{t}$$

Here, *b*_0_ represents the pre-event baseline level, *t* is the predictor *time* (in our example, coded 1–90), and its coefficient, *b*_1,_estimates the trend prior to the event (Wagner et al., 2002, p. 31). The dummy variable *event_t_* codes for whether or not each time point occurred before or after the event (0 for all points prior to the event; 1 for all points after). Its coefficient, *b*_2_, assesses the post-event baseline level (intercept). The variable *t after event* represents how many units after the event the observation took place (0 for all points prior to the event; 1, 2, 3 … for subsequent time points), and its coefficient, *b*_3_, estimates the *change in trend* over the two segments. Therefore, the sum of the pre-event trend (*b*_1_) and its estimated change (*b*_3_) yields the post-event slope (Wagner et al., 2002, p. 301).

Importantly, this analysis requires that the time of event occurrence be specified a priori, otherwise a researcher may search the series in an “exploratory” fashion and discover a time point that yields a notable effect, resulting in potentially spurious results (McCleary et al., 1980, p. 143). In our example, the event of interest was the economic crisis of 2008. However, as is often the case when analyzing large-scale social phenomena, it was not a discrete, singular incident, but rather unfolded over time. Thus, no exact point in time can *perfectly* represent its moment of occurrence. In other topics of psychological research, the event of interest is a unique post-event time may be identified. Although interrupted time series analysis requires that events be discrete, this conceptual problem can be easily managed in practice; selecting a point of demarcation that *generally* reflects when the event occurred will still allow the statistical model to assess the impact of the event on the level and trend of the series. Therefore, due to prior theory and for simplicity, we specified the pre- and post-crisis segments to be separated at January 2008, representing the beginning of the economic crisis and acknowledging that this demarcation was imperfect, but one that would still allow the substantive research question of interest to be answered.

Although not utilized in our analysis, when analyzing an interrupted time series using segmented regression one has the option of actually specifying the post-event segment *after* the actual event occurred. The rationale behind this is to accommodate the time it takes for the causal effect of the event itself manifest in the time series—the *equilibration period* (see Mitchell and James, 2001, p. 539; Wagner et al., 2002, p. 300). Although an equilibration period is likely a component of all causal phenomena (i.e., causal effects probably never fully manifest at once), two prior reviews have illustrated that researchers account for it only infrequently, both theoretically and empirically (Kelly and McGrath, 1988; Mitchell and James, 2001). Statistically, this is accomplished through the segmented regression model above, but simply coding the event as occurring later in the series. Comparing models with different post-event start times can also allow competitive tests of the equilibration period.

#### Empirical example

**Figure 7:**
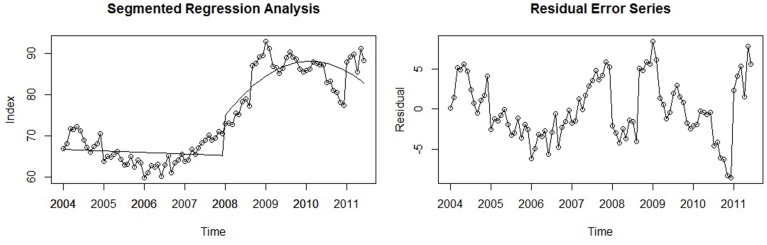
**A segmented regression model used to assess the effect of the 2008 economic crisis on the time series and its associated residual error series**.

For our working example, a segmented regression model was fit to the seasonally adjusted Google time series: A linear trend estimated the first segment and a quadratic trend was fit to the second due to the noted curvilinear form of the second half of the series. Thus, a new variable and coefficient were added to the formal model to account for this non-linearity: *t after event^2^* and *b*_4_, respectively. The results of the analysis indicated that there was a practically significant effect of the crisis: The parameter representing an immediate change in the post-event level was *b*_2_ = 8.66, *p* < 0.001. Although the level (i.e., intercept) differed across segments, the post-crisis *trend* appears to be the most notable change in the series. That is, the real effect of the crisis unfolded over time rather than having an immediately abrupt impact. This is reflected in the other coefficients of the model: The pre-crisis trend was estimated to be near zero (*b*_1_ = −0.03, *p* = 0.44), and the post-crisis trend terms were *b*_3_ = 0.70, *p* < 0.001 for the linear component, and *b*_4_ = −0.02, *p* < 0.001 for the quadratic term, indicating that there was a marked change in trend, but also that it was concave (i.e., on the whole, slowly decreasing over time). Graphically the model seems to capture the underlying trend of both segments exceptionally well (*R*^2^ = 0.87, *p* < 0.001), as the residual error series has almost reached stationarity (*ADF* = −3.38, *p* = 0.06). Both are shown in Figure 7 below.

### Estimating seasonal effects

#### Overview

Up until now, we have chosen to remove any seasonal effects by working with the seasonally adjusted time series in order to more fully investigate a trend of substantive interest. This was consistent with the following adage of time series modeling: When a systematic trend or seasonal pattern is present, it must either be modeled or removed. However, psychological researchers may also be interested in the presence and nature of a seasonal effect, and seasonal adjustment would only serve to remove this component of interest. Seasonality was defined earlier as any regular pattern of fluctuation (i.e., movement up or down in the level of the series) associated with some aspect of the calendar. For instance, although online job searchers exhibited an underlying trend in our data *across* years, they also display the same pattern of movement *within* each year (i.e., across months; see Figure 1). Following the need for more time-based theory and empirical research, seasonal effects are also increasingly recognized as significant for psychological science. In a recent conceptual review Dalal et al. (2014) noted that, “mood cycles… are likely to occur simultaneously over the course of a day (relatively short term) and over the course of a year (long term)” (p. 1401). Relatedly, Larsen and Kasimatis (1990) used time series methods to examine the stability of mood fluctuations across individuals. They uncovered a regular weekly fluctuation that was stronger for introverted individuals than for extraverts (due to the latter's sensation-seeking behavior that resulted in greater mood variability).

Furthermore, many systems of interest exhibit rhythmicity. This can be readily observed across a broad spectrum of phenomena that are of interest to psychological researchers. At the individual level, there is a long history in biopsychology exploring the cyclical pattern of human behavior as a function of biological processes. Prior research has consistently shown that humans possess many common physiological and behavioral cycles that range from 90-min to 365-days (Aschoff, 1984; Almagor and Ehrlich, 1990) and may affect important psychological outcomes. For instance, circadian rhythms are particularly well-known and are associated with physical, mental, and behavioral changes within a 24-h period (McGrath and Rotchford, 1983). It has been suggested that peak motivation levels may occur at specific points in the day (George and Jones, 2000), and longer cyclical fluctuations of emotion, sensitivity, intelligence, and physical characteristics over days and weeks have been identified (for a review, see Conroy and Mills, 1970; Luce, 1970; Almagor and Ehrlich, 1990). Such cycles have been found to affect intelligence test performance and other physical and cognitive tasks (e.g., Latman, 1977; Kumari and Corr, 1996).

#### Regression with seasonal indicators

As previously stated, when seasonal effects are theoretically important, seasonal adjustment is undesirable because it removes the time series component pertinent to the research question at large. An alternative is to qualitatively describe the seasonal pattern or formally specify a regression model that includes a variable which estimates the effect of each season. If a simple linear approximation is used for the trend, the formal model can be expressed as:

(7) $$y_{t}=b_{0}t+b_{1}+\ldots +b_{S}+\varepsilon_{t},$$

where *b*_0_ is now the estimate of the linear relationship between the dependent variable and time, and the coefficients *b*_1:S_ are estimates of the *S* seasonal effects (e.g., *S* = 12 for yearly data; Cowpertwait and Metcalfe, 2009, p. 100). Put more intuitively, this model can still be conceived of as a linear model but with a different estimated intercept for each season that represents its effect (Notice that the *b*_1:S_ parameters are not coefficients but constants).

As an example, the model above was fit to the original, non-seasonally adjusted Google data. Although modeling the series as a linear function of time was found to produce inaccurate forecasts, it can be used when estimating seasonal effects because this component of the model does not affect the estimates of the seasonal effects. For our data, the estimates of each monthly effect were: *b*_1_ = 67.51, *b*_2_ = 59.43, *b*_3_ = 60.11, *b*_4_ = 60.66, *b*_5_ = 63.59, *b*_6_ = 66.77, *b*_7_ = 63.70, *b*_8_ = 62.38, *b*_9_ = 60.49, *b*_10_ = 56.88, *b*_11_ = 52.13, *b*_12_ = 45.66 (Each effect was statistically significant at *p* < 0.001). The pattern of these intercepts mirrors the pattern of movement qualitatively described in the discussion on the seasonal component: Online job search behaviors begin at its highest levels in January (*b*_1_ = 67.51), likely due to the end of holiday employment, and then dropped significantly in February (*b*_2_ = 59.43). Subsequently, its level continued to rise during the next 4 months until June (*b*_6_ = 66.77), after which the series decreased each successive month until reaching its lowest point in December (*b*_12_ = 45.66).

#### Harmonic seasonal models

Another approach to modeling seasonal effects is to fit a *harmonic* seasonal model that uses sine and cosine functions to describe the pattern of fluctuations seen across periods. Seasonal effects often vary in a smooth, continuous fashion, and instead of estimating a discrete intercept for each season, this approach can provide a more realistic model of seasonal change (see Cowpertwait and Metcalfe, 2009, pp. 101–108). Formally, the model is:

(8) $$y_{t}=m_{t}+\sum_{i=1}^{S/2}\left[s_{i}\sin(2\pi it/S)+c_{i}\cos(2\pi it/S)\right]+\varepsilon_{t},$$

where *m_t_* is the estimate of the trend at *t* (approximated as a linear or polynomial function of time), *s_i_* and *c_i_* are the unknown parameters of interest, *S* is the number of seasons within the time period (e.g., 12 months for a yearly period), *i* is an index that ranges from 1 to *S/2*, and *t* is a variable that is coded to represent time (e.g., 1:90 for 90 equally-spaced observations). Although this model is complex, it can be conceived as including a predictor for each season that contains a sine and/or cosine term. For yearly data, this means that six *s* and six *c* coefficients estimate the seasonal pattern (*S/2* coefficients for each parameter type). Importantly, after this initial model is estimated, the coefficients that are not statistically significant can be dropped, which often results in fewer parameters relative to the seasonal indicator model introduced first (Cowpertwait and Metcalfe, 2009, p. 104). For our data, the above model was fit using a linear approximation for the trend, and five of the original twelve seasonal coefficients were statistically significant and thus retained: *c*_1_ = −5.08, *p* < 0.001, *s*_2_ = 2.85, *p* = 0.005, *s*_3_ = 2.68, *p* = 0.009, *c*_3_ = −2.25, *p* = 0.03, *c*_5_ = −2.97, *p* = 0.004. This model also explained a substantial amount of the series variance (*R*^2^ = 0.75, *p* < 0.001). Pre-made and annotated R code for this analysis can be found in the Supplementary Material.

## Time series forecasting: ARIMA (*p, d, q*) modeling

In the preceding section, a number of descriptive and explanatory regression models were introduced that addressed various topics relevant to psychological research. First, we sought to determine how the trend in the series could be best described as a function of time. Three models were fit to the data, and modeling the trend as a cubic function provided the best fit: It was the most parsimonious model that explained a very large amount of variation in the series, it did not systematically over or underestimate many successive observations, and any potential forecasts were clearly superior relative to those of the simpler linear and quadratic models. In the subsequent section, a segmented regression analysis was conducted in order to examine the impact of the 2008 economic crisis on job search behavior. It was found that there was both a significant immediate increase in the baseline level of the series (intercept) and a concomitant increase in its trend (i.e., slope) that gradually decreased over time. Finally, the seasonal effects of online search behavior were estimated and mirrored the pattern of job employment rates described in a prior section.

From these analyses, it can be seen that the main features of many times series are the trend and seasonal components that must either be modeled as deterministic functions of predictors or removed from the series. However, as previously described, another critical feature in time series data is its *autocorrelation*, and a large portion of time series methodology is aimed at explaining this component (Dettling, 2013, p. 2). Primarily, accounting for autocorrelation entails fitting an ARIMA model to the original series, or adding ARIMA terms to a previously fit regression model; ARIMA models are the most general class of models that seek to explain the autocorrelation frequently found in time series data (Hyndman and Athanasopoulos, 2014). Without these terms, a regression model will ignore the pattern of autocorrelation among the residuals and produce less accurate forecasts (Hyndman and Athanasopoulos, 2014). Therefore, ARIMA models are predictive *forecasting models*. Time series models that include both regression and ARIMA terms are referred to as *dynamic* models and may be a primary type of time series models used by psychological researchers.

Although not strongly emphasized within psychological science, forecasting is an important aspect of scientific verification (Popper, 1968). Standard cross-sectional and longitudinal models are generally used in an explanatory fashion (e.g., estimating the relationships among constructs and testing null hypotheses), but they are quite capable of prediction as well. Because of the ostensible movement to more time-based empirical research and theory, predicting future values will likely become a more important aspect of statistical modeling, as it can validate psychological theory (Weiss and Cropanzano, 1996) and computational models (Tobias, 2009) that specify effects over time.

At the outset, it is helpful to note that the regression and ARIMA modeling approaches are not substantially different: They both formalize the variation in the time series variable as a function of predictors and some stochastic noise (i.e., the error term). The only practical difference is that while regression models are generally built from prior research or theory, ARIMA models are developed *empirically* from the data (as will be seen presently; McCleary et al., 1980, p. 20). In describing ARIMA modeling, the following sections take the form of those discussing regression methods: Conceptual and mathematical treatments are provided in complement in order to provide the reader with a more holistic understanding of these methodologies.

### Introduction

The first step in ARIMA modeling is to visually examine a plot of the series' ACF (autocorrelation function) to see if there is any autocorrelation present that can be used to improve the regression model—or else the analyst may end up adding unnecessary terms. The ACF for the Google data is shown in Figure 3. Again, we will work with the seasonally adjusted series for simplicity. More formally, if a regression model has been fit, the *Durbin–Watson* test can be used to assess if there is autocorrelation among the residuals and if ARIMA terms can be included to improve its forecasts. The Durbin–Watson test tests the null hypothesis that there is no lag-1 autocorrelation present in the residuals. Thus, a rejection of the null means that ARIMA terms can be included (the Ljung–Box test described below can also be used; Hyndman and Athanasopoulos, 2014).

Although the modeling techniques described in the present and following sections can be applied to any one of these models, due to space constraints we continue the tutorial on time series modeling using the cubic model of the first section. A model with only one predictor (viz., time) will allow more focus on the additional model terms that will be added to account for the autocorrelation in the data.

### I(*d*): integrated

#### Overview

ARIMA is an acronym formed by the three constituent parts of these models. The AR(*p*) and MA(*q*) components are predictors that explain the autocorrelation. In contrast, the *integrated* (I[*d*]) portion of ARIMA models does not add predictors to the forecasting equation. Rather, it indicates the order of differencing that has been applied to the time series in order to remove any trend in the data and render it stationary. Before any AR or MA terms can be included, *the series must be stationary*. Thus, ARIMA models allow non-stationary series to be modeled due to this “integrated” component (an advantage over simpler ARMA models that do not include such terms; Cowpertwait and Metcalfe, 2009, p. 137). A time series that has been made stationary by taking the *d* difference of the original series is notated as I(*d*). For instance, an I(1) model indicates that the series that has been made stationary by taking its first differences, I(2), by the second differences (i.e., the first differences of the first differences), etc. Thus, the order of integrated terms in an ARIMA model merely specifies how many iterations of differencing were performed in order to make the series stationary so that AR and MA terms may be included.

#### Identifying the order of differencing

Identifying the appropriate order of differencing to stationarize the series is the first and perhaps most important step in selecting an ARIMA model (Nua, 2014). It is also relatively straightforward. As stated previously, the order of differencing rarely needs to be greater than two in order to stationarize the series. Therefore, in practice the choice comes down to whether the series is transformed into either its first or second differences, the optimal choice being the order of differencing that results in the lowest series variance (and does not result in an increase in variance that characterizes overdifferencing).

### AR(*p*): autoregressive terms

#### Overview

The first part of an ARIMA model is the AR(*p*) component, which stands for *autoregressive*. As correlation is to regression, autocorrelation is to autoregression. That is, in regression, variables that are correlated with the criterion can be used for prediction, and the model specifies the criterion as a function of the predictors. Similarly, with a variable that is autocorrelated (i.e., correlated with itself across time periods), past values can serve as predictors, and the values of the time series are modeled as a function of previous values (thus, *autoregression*). In other words, an ARIMA (*p, d, q*) model with *p* AR terms is simply a linear regression of the time series values against the preceding *p* observations. Thus, an ARIMA(1, *d, q*) model includes one predictor, the observation immediately preceding the current value, and an ARIMA(2, *d, q*) model includes two predictors, the first and second preceding observations. The number of these autoregressive terms is called the *order* of the AR component of the ARIMA model. The following equation uses one AR term (an AR[1] model) in which the preceding value in the time series is used as a regressor:

(9) $$y_{t}=\phi (y_{t-1})+\varepsilon_{t},$$

where ϕ is the autoregressive coefficient (interpretable as a regression coefficient), and *y_t−1_* is the immediately preceding observation. More generally, a model with AR(*p*) terms is expressed as:

(10) $$y_{t}=\phi_{1}\left(y_{t-1}\right)+\phi_{2}\left(y_{t-2}\right)+\ldots +\phi_{p}\left(y_{t-p}\right)+\varepsilon_{t}.$$

#### Selecting the number of autoregressive terms

The number of autoregressive terms required depends on how many lagged observations explain a significant amount of *unique* autocorrelation in the time series. Again, an analogy can be made to multiple linear regression: Each predictor should account for a significant amount of variance after controlling for the others. However, a significant autocorrelation at higher lags may be attributable to an autocorrelation at a lower lag. For instance, if a strong autocorrelation exists at lag-1, then a significant lag-3 autocorrelation (i.e., a correlation of time *t* with *t*-3) may be a result of *t* being correlated with *t*-1, *t*-1 with *t*-2, and *t*-2 with *t*-3 (and so forth). That is, a strong autocorrelation at an early lag can “persist” throughout the time series, inducing significant autocorrelations at higher lags. Therefore, instead of inspecting the ACF which displays zero-order autocorrelations, a plot of the *partial autocorrelation function* (PACF) across different lags is the primary method in determining which prior observations explain a significant amount of *unique* autocorrelation, and accordingly, how many AR terms (i.e., lagged observations as predictors) should be included. Put simply, the PACF displays the autocorrelation of each lag after controlling for the autocorrelation due to all preceding lags (McCleary et al., 1980, p. 75). A conventional rule is that if there is a sharp drop in the PACF after *p* lags, then the previous *p*-values are responsible for the autocorrelation in the series, and the model should include *p* autoregressive terms (the partial autocorrelation coefficient typically being the value of the autoregressive coefficient, ϕ; Cowpertwait and Metcalfe, 2009, p. 81). Additionally, the ACF of such a series will gradually decay (i.e., reduce) toward zero as the lag increases.

**Figure 8:**
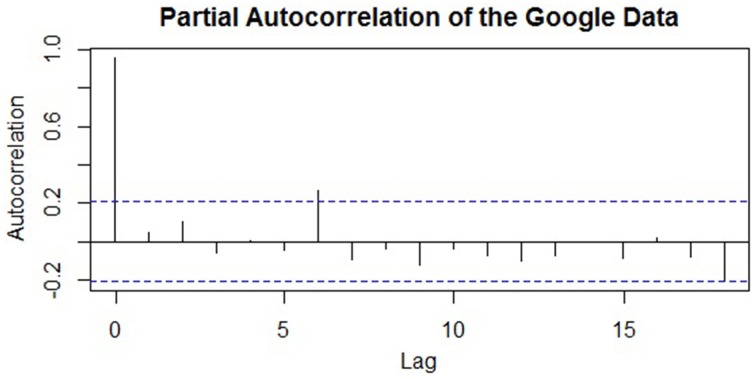
**A plot of the partial autocorrelation function (PACF) of the seasonally adjusted time series of Google job searches**.

Applying this knowledge to the empirical example, Figure 3 depicted the ACF of the seasonally adjusted Google time series, and Figure 8 displays its PACF. Here, only one lagged partial autocorrelation is statistically significant (lag-6), despite over a dozen autocorrelations in the ACF reaching significance. Thus, it is probable that early lags—and the lag-6 in particular—are responsible for the chain of autocorrelation that persists throughout the series. Although the series is considerably non-stationary (i.e., there is a marked trend and seasonal component), if the series was already stationary, then a model with a single AR term (an AR[1] model) would likely provide the best fit, given a single significant partial autocorrelation at lag-6. The ACF in Figure 3 also displays the characteristics of an AR(1) series: It has many significant autocorrelations that gradually reduce toward zero. This coheres with the notion that one AR term is often sufficient for a residual time series (Cowpertwait and Metcalfe, 2009, p. 121). However, if the pattern of autocorrelation is more complex, then additional AR terms may be required. Importantly, if a particular number of AR terms have been successful in explaining the autocorrelation of a stationary series, the residual error series should appear as entirely random white noise (as in Figure 4).

### MA(*q*): moving average terms

#### Overview

In the preceding section, it was shown that one can account for the autocorrelation in the data by regressing on prior values in the series (AR terms). However, sometimes the autocorrelation is more easily explained by the inclusion of MA terms; the use of MA terms to explain the autocorrelation—either on their own or in combination with AR components—can result in greater *parameter parsimony* (i.e., fewer parameters), relative to relying solely on AR terms (Cowpertwait and Metcalfe, 2009, p. 127). As noted above, ARIMA models assume that any systematic components have either been modeled or removed and that the time series is stationary—i.e., a stochastic process. In time series theory, the values of stochastic processes are determined by two forces: prior values, described in the preceding section, and *random shocks* (i.e., errors; McCleary et al., 1980, pp. 18–19). Random shocks are the myriad variables that vary across time and interact with such complexity that their behavior is ostensibly random (e.g., white noise; McCleary et al., 1980, p. 40). Each shock can be conceived of as an *unobserved* value at each point in time that influences each *observed* value of the time series. Thus, autocorrelation in the data may be explained by the persistence of prior values (or *outputs*, as in AR terms) or, alternatively, the lingering effects of prior unobserved shocks (i.e., the *inputs*, in MA terms). Therefore, if prior random shocks are related to the value of the series, then these can be included in the prediction equation to explain the autocorrelation and improve the efficiency of the forecasts generated by the model. In other words, just as AR terms can be conceived as a linear regression on previous time series values, MA terms are conceptually a linear regression of the current value of the series against prior random shocks. For instance, an MA(1) model can be expressed as:

(11) $$y_{t}=\theta \left(\varepsilon_{t-1}\right)+\varepsilon_{t},$$

where ε*_t_* is the value of the random shock at time *t, ε_t−_*_1_ is the value of the previous random shock, and *θ* is its coefficient (again, interpretable as a regression coefficient). More generally, the order of MA terms is conventionally denoted as *q*, and an MA(*q*) model can be expressed as:

(12) $$y_{t}=\theta_{1}\left(\varepsilon_{t-1}\right)+\theta_{2}\left(\varepsilon_{t-2}\right)+\ldots +\theta_{q}\left(\varepsilon_{t-q}\right)+\varepsilon_{t}.$$

#### Selecting the number of MA terms

Selecting the number of MA terms in the model is conceptually similar to the process of identifying the number of AR terms: One examines plots of the autocorrelation (ACF) and partial autocorrelation functions (PACF) and then specifies an appropriate model. However, while the number of AR terms could be identified by the PACF of the series (more specifically, the point at which the PACF dropped), the number of appropriate MA terms is usually identified by the ACF. Specifically, if the ACF is non-zero for the first *q* lags and then drops toward zero, then *q* MA terms should be included in the model (McCleary et al., 1980, p. 79). All successive lags of the ACF are expected to be zero, and the PACF of such a series will be gradually decaying (McCleary et al., 1980, p. 79). Thus, relative to AR terms, the roles of the ACF and PACF are essentially *reversed* when determining the number of MA terms. Furthermore, in practice most social processes can be sufficiently modeled by a single MA term; models of order *q* = 2 are less common, and higher-order models are extremely rare (McCleary et al., 1980, p. 63).

### Model building and further notes on ARIMA (*p, d, q*) models

The components of ARIMA models—autoregressive, integrated, and moving average—are aimed at explaining the autocorrelation in a series that is either stationary or can be made so through differencing (i.e., I[*d*] integrated terms). Though already stated, the importance of the following point warrants reiteration: After a successful ARIMA(*p, d, q*) model has been fit to the autocorrelated data, the residual error series should be a white noise series. That is, after a good-fitting model has been specified, the residual error series should not display any significant autocorrelations, have a mean of zero, and some constant variance; i.e., there should be no remaining signal that can be used to improve the model's forecasts. Thus, after specifying a particular model, visual inspection of the ACF and PACF of the error series is critical in order to assess model adequacy (McCleary et al., 1980, p. 93). All autocorrelations are expected to be zero with 5% expected to be statistically significant due to sampling error.

Furthermore, just as there are formal methods to test that a series is stationary before fitting an ARIMA model, there are also statistical tests for the presence of autocorrelation after the model has been fit. The Ljung–Box test (Ljung and Box, 1978) is one commonly-applied method in which the null hypothesis is that the errors are uncorrelated across many lags (Cryer and Chan, 2008, p. 184; Hyndman and Athanasopoulos, 2014). Thus, failing to reject the null provides evidence that the model has succeeded in explaining the remaining autocorrelation in the data.

If both formal and informal methods indicate that the residual error series is not a series of white noise terms (i.e., there is remaining autocorrelation), then the analyst must reassess the pattern of autocorrelation and re-specify a new model. Thus, in contrast to regression approaches, ARIMA modeling is an exploratory, iterative process in which the data is examined, models are specified, checked for adequacy, and then re-specified as needed. However, selecting the most appropriate order of AR, I, and MA terms can prove to be a difficult process (Hyndman and Athanasopoulos, 2014). Fortunately, model comparison can be easily performed by comparing the Akaike information criterion (AIC) across models (Akaike, 1974)^7^. This statistic is based on the fit of a model and its number of parameters, and models with lower values should be selected. Generally, models within two AIC values are considered comparable, a difference of 4–7 points indicates considerable support for the better-fitting model, and a difference of 10 points or greater signifies full support of that model (Burnham and Anderson, 2004, p. 271). Additionally, the “forecast” R package (Hyndman, 2014) contains a function to automatically derive the best-fitting ARIMA model based on the AIC or other fit criteria (see Hyndman and Khandakar, 2008). This procedure is discussed in the Supplementary Material.

Furthermore, a particular pattern of autocorrelation can often be explained by *either* AR or MA terms. Generally, AR terms are preferable to MA terms because their interpretation of these parameters is more straightforward (e.g., the regression coefficient associated with a previous time series value rather than a coefficient associated with an unobserved random shock). However, a more central concern is parameter parsimony; if a model using MA terms (or a combination of AR and MA terms) can explain the autocorrelation with fewer parameters than one that relies solely on AR terms, then these models are generally preferable.

Finally, although a mixed ARIMA model containing both AR and MA terms can result in greater parameter parsimony (Cowpertwait and Metcalfe, 2009, p. 127), in practice, non-mixed models (i.e., those with either with AR or MA terms alone) should always be ruled out prior to fitting these more complex models (McCleary et al., 1980, p. 66). Unnecessary model complexity (i.e., redundant parameters) may not become evident at all during the process of model checking, while the inadequacy of simpler models is often easily identified (e.g., noticeable remaining autocorrelation in the ACF plot).

## Fitting a dynamic regression model with ARIMA terms

In this final section, we illustrate how a predictive ARIMA approach to time series modeling can be combined with regression methods through specification of a dynamic regression model. These models can be fit to the data in order to generate accurate forecasts, as well as explain or examine an underlying trend or seasonal effect (as opposed to their removal). We then analyze the predictions from this model and discuss methods of assessing forecast accuracy. For simplicity, we continue with the regression model that modeled the series as a cubic function of time.

### Preliminaries

When the predictor is time, one should begin specification of a dynamic regression model by first examining the residual error series after the regression model has been fit. This is done in order to first detect if there is any autocorrelation in the model residuals that would warrant the inclusion of ARIMA terms. The residual error series are of interest because a dynamic regression model can be thought of as a hybrid model that includes a correction for autocorrelated errors. That is, whatever the regression model does not account for (trend, autocorrelation, etc.) can be supplemented by ARIMA modeling. Analytically, this is performed by re-specifying the initial regression model as an ARIMA model with regressors (sometimes called an “ARIMAX” model, the “X” denoting external predictors) and selecting the appropriate order of ARIMA terms that fit the autocorrelation structure in the residuals (Nua, 2014).

### Identifying the order of differencing: I(*d*) terms

As noted previously, the residual error series of the cubic regression model exhibited a remaining trend and autocorrelation (see Figure 6). A significant Durbin–Watson test formally confirms this is the case (i.e., the error terms are not uncorrelated; *DW* = 0.47, *p* < 0.001). Thus, ARIMA terms are necessary to (a) stationarize the series (I[*d*] terms) and (b) generate more accurate forecasts (AR[*p*] and/or MA[*q*] terms). As stated above, the conventional first step when formulating an ARIMA is determining the number of I(*d*) terms (i.e., order of differencing) required to remove any remaining trend and render the series stationary. We note that, in this case, the systematic seasonal effects have already been removed through seasonal adjustment. It was previously noted that in practice, removing a trend is accomplished almost always by taking either the first or second differences—whichever transformation results in the lowest variance and avoids overdifferencing (i.e., an *increase* in the series variance). Because the residual trend does not have a markedly changing slope, it is likely that only one order of differencing will be required. The results indicate that this is indeed the case: After first differencing, the series variance is reduced from 13.56 to 6.45, and an augmented Dickey–Fuller test rejects the null hypothesis of a non-stationary series (*ADF* = −4.50, *p* < 0.01). Taking the second differences also results in stationarity (i.e., the trend is removed), but leads to an overdifferenced series with a variance that is inflated to a level higher than the original error series (*s*^2^ = 14.90).

### Identification of AR(*p*) and MA(*q*) terms

**Figure 9:**
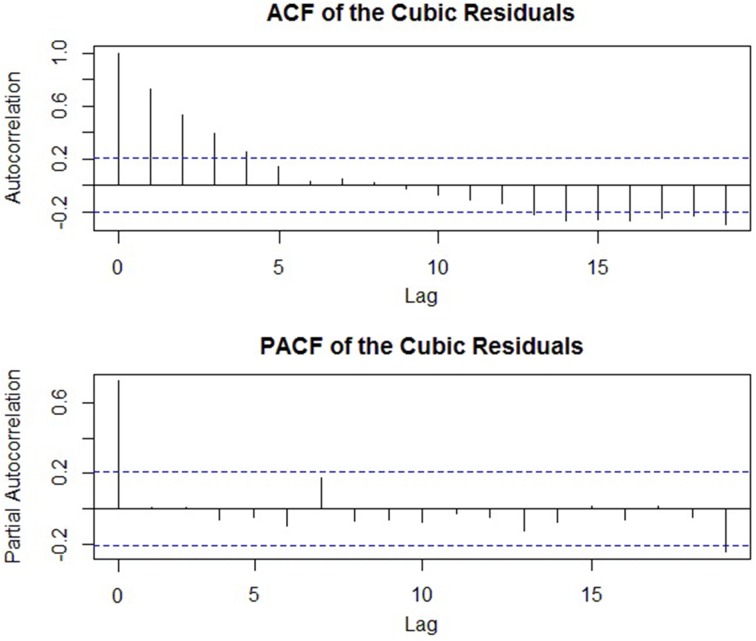
**ACF and PACF of the cubic model residuals used to determine the number of AR and MA terms in an ARIMA model**.

After the order of I(*d*) terms has been identified (here, 1), the next step is to determine whether the pattern of autocorrelation can be better explained by AR terms, MA terms, or a combination of both. As noted, AR terms are often preferred to MA terms because their interpretation is more straightforward, and simpler models with either AR or MA terms are preferable to mixed models. We therefore begin by examining plots of the ACF and PACF for the residual error series shown Figure 9 in order to see if they display either an AR or MA “signature” (e.g., drop-offs or slow decays).

From Figure 9, we can see that there are many high autocorrelations in the ACF plot that slowly decay, indicative that AR terms are probably most suitable (A sharp drop in the ACF would indicate that the autocorrelation is probably better explained by MA terms). As stated earlier, the PACF gives the autocorrelation for a lag after controlling for all earlier lags; a significant drop in the PACF at a particular lag indicates that this lagged value is largely responsible for the large zero-order autocorrelations in the ACF. Based on this PACF, the number of terms to include is less clear; aside from the lag-0 autocorrelation, there is no perceptible drop-off in the PACF, and there are no strong partial autocorrelations to attribute the persistence of the autocorrelation seen in the ACF. However, we know that there is autocorrelation in the model residuals, and that either one or two AR terms are typically sufficient for accounting for any autocorrelation (Cowpertwait and Metcalfe, 2009, p. 121). Therefore, we suspect that a single AR term can account for it. After fitting an ARIMA (1, 1, 0) model, a failure to reject the null hypothesis in a Ljung–Box test indicated that the model residuals were indistinguishable from a random white noise series (*χ*^2^ = 0.005, *p* = 0.94), and less than 5% of the autocorrelations in the ACF were statistically significant (The AIC of this model was 419.80). For illustrative purposes, several other models were fit to the data that either included additional AR or MA terms, or a combination of both. Their relative fit was analyzed and the results are shown in Table 2. As can be seen, the ARIMA (1, 1, 0) model provided a level of fit that exceeded all of the other models (i.e., the smallest AIC difference among models was 4, showing considerable support). Thus, this model parsimoniously accounted for the systematic trend through a combination of regression modeling and first differencing and successfully extracted all the autocorrelation (i.e., signal) from the data in order to achieve more efficient forecasts.

**Table 2:** **Comparison of different ARIMA models**.

**Model**	**Residual analysis**	**AIC**
ARIMA(1, 1, 0): one AR term	Ljung–Box test: *χ*^2^ = 0.005, *p* = 0.94	419.80
ARIMA(0, 1, 1): one MA term	Ljung–Box test: *χ*^2^ = 0.01, *p* = 0.92	423.84
ARIMA(1, 1, 1): a mixed model	Ljung–Box test: *χ*^2^ = 0.02, *p* = 0.89	425.84
ARIMA(2, 1, 0): two AR terms	Ljung–Box test: *χ*^2^ = 0.61, *p* = 0.43	448.79
ARIMA(0, 1, 2): two MA terms	Ljung–Box test: *χ*^2^ = 0.02, *p* = 0.89	425.84

*ACF plots for all models showed that <5% of autocorrelations reached statistical significance*.

### Forecasting methods and diagnostics

Because forecasts into the future cannot be directly assessed for accuracy until the actual values are observed, it is important that the analyst establish the adequacy of the model prior to forecasting. To do this, the analyst can partition the data into two parts: the *estimation period*, comprising about 80% of the initial observations and used to estimate the model parameters, and the *validation period*, usually about 20% of the data and used to ensure that the model predictions are accurate. These percentages may shift depending on the length of the series (see Nua, 2014), but the size of the validation period should at least equal the number of periods ahead the analyst wishes to forecast (Hyndman and Athanasopoulos, 2014). The predictions generated by the model are then compared to the observed data in the validation period to assess their accuracy. Evaluating forecast accuracy is accomplished by examining the residuals for any systematic patterns of misspecification. Forecasts should ideally be located within the 95% confidence limits, and formal statistics can be calculated from the model residuals in order to evaluate its adequacy. A popular and intuitive statistic is the *mean absolute error* (MAE): the average absolute deviation from the predicted values. However, this value cannot be used to compare models, as it is scale-dependent (e.g., a residual with an absolute value of 10 is much less egregious when forecasting from a series whose mean is 10,000 relative to a series with a mean of 10). Another statistic, the *mean absolute percentage error* (MAPE) is useful for comparing across models and is defined as the average percentage that the forecasts deviated from the observed values. Other methods and statistics, such as the *root mean squared error* (RMSE) and the *mean absolute scaled error* (MASE) can aid model evaluation and selection and are accessibly discussed by Hyndman and Athanasopoulos (2014, chap. 2). Once a forecasting model has been deemed sufficiently accurate through these methods, forecasts into the future can then be calculated with relative confidence.

**Figure 10:**
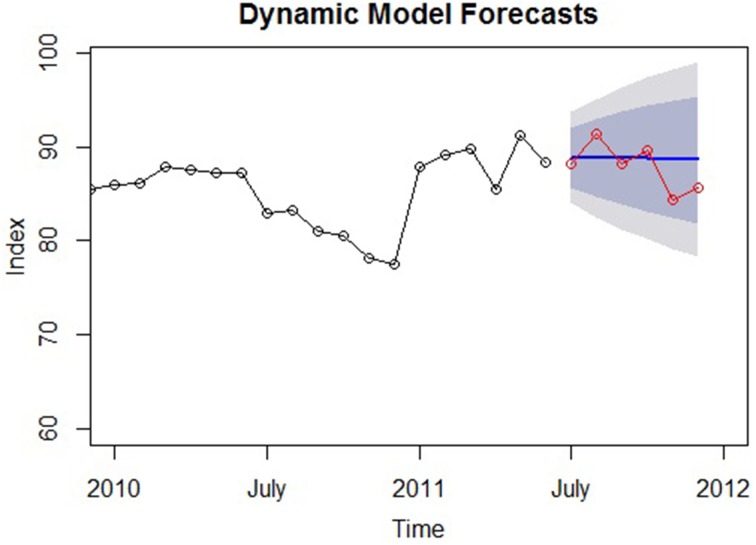
**Forecasts from the dynamic regression model compared to the observed values**. The blue line represents the forecasts, and the red dotted line indicates the observed values. The darker gray region denotes the 80% confidence region, the lighter gray, 90%.

Because we have the benefit of hindsight in our example, all observations were used for estimation, and six forecasts were generated for the remainder of the 2011 year and compared to the actual observed values. The point forecasts (blue line), 80%, and 95% confidence limits are displayed in Figure 10 juxtaposed against the actual values in red. As can be seen, this forecasting model is generally successful: Each observed value lies within the 80% limits, and the residuals have a low mean absolute error (*MAE* = 2.03) relative to the series mean (*M* = 75.47), as well as a low mean absolute percentage error (*MAPE* = 2.33). Additional statistics verified the accuracy of these predictions, and the full results of the analysis can be obtained from the first author.

As a final note on ARIMA modeling, if the sole goal of the analysis is to produce accurate forecasts, then the seasonal and trend components represent a priori barriers to this goal and should be removed through seasonal adjustment and the I(*d*) terms of an appropriate ARIMA model, respectively. Such predictive models are often easier to implement, as there are no systematic components of interest to describe or estimate; they are simply removed through transformations in order to achieve a stationary series. Finally, we close this section with two tables. The first, Table 3, compiles the general steps involved in ARIMA time series modeling described above, from selecting the optimal order of ARIMA terms to assessing forecast accuracy. The second, Table 4, provides a reference for the various time series terms introduced in the current paper.

**Table 3:** **Steps for specifying an ARIMA forecasting model**.

**Specific steps**	**Intended purpose**	**Procedure**
Step 1. Confirm the presence of autocorrelation.	If there is autocorrelation in the data, then an ARIMA model can be used for forecasting or ARIMA terms can be included within an existing regression model to improve its forecast accuracy (i.e., a dynamic regression/ARIMAX model).	• Examine a plot of the ACF for any large autocorrelations across different lags. In a white noise series, 5% of autocorrelations are expected to reach statistical significance, so one must look at strength of the autocorrelation in addition to statistical significance for the best diagnosis. • If a regression model has been fit to the data, one can formally test for a lag-1 autocorrelation in the residuals by conducting a Durbin–Watson or Ljung–Box test (see Table 1).
Step 2. Determine if the series is stationary.	Before AR or MA terms can be included in the model to account for the autocorrelation, the series must be stationary (i.e., a constant mean, variance, and autocorrelation).	• Examine a plot of the series for systematic changes in its mean level (i.e., trend or seasonal effects) and variance. • Conduct an ADF test to formally test for stationarity.
Step 3. Transform the series to stationarity.	AR and MA terms assume a stationary series, and this assumption must be met before modeling the autocorrelation.	• If the variance is not constant over time, taking the natural logarithm of the series can stabilize it. • Seasonal effects can be removed through seasonal adjustment. • A trend component can be removed through differencing and nearly always through either its first or second differences (I[1] or I[2] terms in an ARIMA model, respectively). Each successive order of differencing should further remove the trend and reduce the overall series variance. (But be careful to avoid overdifferencing the series, indicated by an increase in its variance.) • Confirm the series is stationary by performing an ADF test.
Step 3. Partition the data into estimation validation periods.	Before a forecasting model is used, its accuracy should be assessed. This entails conserving some data in the latter portion of the series to compare to the predictions generated by the model (the validation period). However, the majority of the data should still be used for parameter estimation.	• As a general rule, the first 80% of the series can be used to estimate the parameters and the remaining 20% to assess the accuracy of the model predictions. • For longer series, a larger percentage can be used for the validation period, and its size should be at least as large as the number of periods forecasted ahead.
Step 4. Examine the ACF and PACF, and fit a parsimonious ARIMA model.	Examining the ACF and PACF of a series can indicate how many AR and MA terms will be required to explain the series autocorrelation.	• A pattern of autocorrelation that is best explained by AR terms has a steadily decaying ACF and a PACF that drops after *p* lags. If this is the case, then *p* AR terms will generally be required. • If the autocorrelation displays an MA signature (a drop-off in the ACF after *q* lags and a gradually decaying PACF) then a model with *q* MA terms will likely provide the best fit to the data. • Ordinarily, only one or two AR or MA terms are required for explaining a series' autocorrelation.
Step 5. Examine model sufficiency.	A successful model will have extracted all of the autocorrelation from the data after being fit. Noticeable remaining autocorrelation indicates that the model can be improved.	• Examine a plot of the model residuals which should appear as random white noise. • Conduct a Ljung–Box test on the residuals to formally assess if the autocorrelations are significantly different than those expected from a white noise series.
Step 6. Re-specify the model if necessary and use the AIC to compare models.	An initial model may not successfully explain all the autocorrelation present in the data. Alternatively, a model may successfully account for the autocorrelation but be needlessly complex (i.e., more AR or MA terms than is necessary). Thus, ARIMA modeling is an iterative, exploratory process where multiple models are specified and then compared.	• Sometimes a mixed model can explain the autocorrelation using less parameters. Alternatively, a simpler model may also fit the data well. These models can be specified and checked for adequacy (Step 5). • Among the fitted models, compare the AIC which evaluates the fit of the model and includes penalties for model complexity. Models with a smaller AIC value indicate a superior relative fit. • As a rule of thumb, models within two AIC points are comparable, a difference of 4–7 points indicates considerable support, and a difference of 10 points or greater signifies full support.
Step 7. Generate predictions and compare to observations in the validation period.	Once a model has been chosen, comparing the model predictions within the validation period allows the analyst to determine if the model produces accurate forecasts during the time periods that have been observed. This provides evidence that it will provide accurate *future* forecasts whose precision cannot be immediately evaluated.	• After estimating model parameters from the first portion of the data, use the remaining observations to compare to the predicted values given by the model. • Observed values should ideally be located within the 95% confidence limits of the forecasts. • Calculate statistics that quantify its accuracy, such as the MAE and MAPE.
Step 8. Generate forecasts into the future.	After a good-fitting model has been selected and checked for forecasting accuracy, it can be used to generate forecasts into the future.	• Determine how many periods ahead into the future to forecast. • ARIMA models can provide accurate forecasts several periods into the future, but long-term forecasting is inherently more uncertain.

*ACF, Autocorrelation function; PACF, Partial autocorrelation function; ADF, augmented Dickey–Fuller; AIC, Akaike information criterion*.

**Table 4:** **Glossary of time series terms**.

**Term**	**Description**	**Relevance to time series analysis**
Trend	The overarching long-term change in the mean level of a time series.	Trends often represent time series effects that are theoretically interesting, such as the result of a critical event or the effect of other variables. Importantly, trends may be either deterministic or stochastic. Deterministic trends are those due to the constant effects of a few causal forces. As a result, they are generally stable across time and are suitable to be modeled through regression. In contrast, stochastic trends arise simply by chance and are consequently not suitably modeled through regression methods.
Seasonality	A pattern of rises and falls in the mean level of a series that consistently occurs across time periods.	Seasonal effects may be substantively interesting (in which case they should be estimated) or they may obscure other more important components, such as a trend (in which case they should be removed).
Cycles	Any repeating pattern in the mean level of a series whose duration is not fixed or known and generally occurs over a period of 2 or more years.	Cycles may also represent patterns of interest. However, cycles are more difficult to identify and generally require longer series to be adequately captured.
Autocorrelation	When current observations exhibit a dependence upon prior states, manifesting statistically as a correlation between lagged observations.	The presence of autocorrelation means that there is signal in the data that can be modeled by AR or MA terms to generate more accurate forecasts.
Stationarity	When the mean, variance, and autocorrelation of a series are constant across time.	Descriptive statistics of a time series are only meaningful when it is stationary. Furthermore, before a time series can be modeled by AR or MA terms it must be made stationary.
Seasonal adjustment	A process of estimating the seasonal effects and removing them from the series.	Seasonal adjustment can remove a source of variation that is not interesting from a theoretical perspective so that the elements of a time series that are of interest can be more clearly analyzed (e.g., a trend).
Differencing	The process of transforming the values of a series into a series of the differences between observations adjacent in time.	Differencing removes the trend from a time series and thus helps to make the mean of a time series stationary.
Autocorrelation function (ACF)	A measure of linear association (correlation) between the current time series values with its past series values.	The ACF allows the analyst to see if there is any autocorrelation in the data and at what lags it manifests. It is essential in identifying the appropriate number of AR and MA terms to explain the pattern of the residuals. It is also valuable for determining if there is any remaining autocorrelation after an ARIMA model has been fit (i.e., model diagnostics).
Partial autocorrelation function (PACF)	A measure of linear association (correlation) between the current time series values with its past series values after controlling for the intervening observations.	The PACF is useful for identifying the number of AR or MA terms that will explain the autocorrelation in the data.
Integrated (I)	In an ARIMA model, the number of times the series has been differenced in order to make it stationary.	Stationarity is an assumption that must be met before any AR or MA terms can be included in a model. In an ARIMA model, the Integrated component allows the inclusion of series that are non-stationary in the mean.
Autoregressive (AR)	When a variable is regressed on its prior values in order to account for autocorrelation.	AR terms are able to account for autocorrelation in the data to improve forecasts.
Moving average (MA)	When a variable is regressed on past random shocks (error terms) in order to account for autocorrelation.	MA terms are able to account for autocorrelation in the data to improve forecasts.
Dynamic regression (ARIMAX)	A time series model that includes both regression and ARIMA terms.	A model that includes both explanatory variables and AR or MA terms be used to simultaneously model an underlying trend and generate accurate forecasts.

## Addendum: further time series techniques and resources

Finally, because time series analysis contains a wide range of analytic techniques, there was not room to cover them all here (or in any introductory article for that matter). For a discussion of computing correlations between time series (i.e., the cross-correlation function), the reader is directed to McCleary et al. (1980). For a general introduction to regression modeling, Cowpertwait and Metcalfe (2009) and Ostrom (1990) have excellent discussions, the latter describing the process of identifying lagged effects. For a highly accessible exposition of identifying and cycles or seasonal effects within the data through *periodogram* and *spectral analysis*, the reader should consult Warner (1998), a social scientist-based text which also describes *cross-spectral analysis*, a method for assessing how well cycles within two series align. For regression modeling using other time series as substantive predictors, the analyst can use *transfer function* or dynamic regression modeling and is referred to Pankratz (1991) and Shumway and Stoffer (2006) for further reading. For additional information on forecasting with ARIMA models and other methods, we refer the reader to Hyndman and Athanasopoulos (2014) and McCleary et al. (1980). Finally, multivariate time series analysis can model reciprocal causal relations among time series in a modeling technique called *vector* ARMA models, and for discussions we recommend Liu (1986), Wei (2006), and the introduction in Pankratz (1991, chap. 10). Future work should attempt to incorporate these analytic frameworks within psychological research, as the analysis of time series brings in a host of complex issues (e.g., detecting cycles, guarding against spurious regression and correlation) that must be handled appropriately for proper data analysis and the development of psychological theory.

## Conclusion

Time series analysis has proved to be integral for many disciplines over many decades. As time series data becomes more accessible to psychologists, these methods will be increasingly central to addressing substantive research questions in psychology as well. Indeed, we believe that such shifts have already started and that at an introduction to time series data is substantially important. By integrating time-series methodologies within psychological research, scholars will be impelled to think about how variables at various psychological levels may exhibit trends, cyclical or seasonal patterns, or a dependence on prior states (i.e., autocorrelation). Furthermore, when examining the influence of salient events or “shocks,” essential questions, such as “What was the pre-event trend?” and “How long did its effects endure, and what was its trajectory?” will become natural extensions. In other words, researchers will think in an increasingly *longitudinal* manner and will possess the necessary statistical knowledge to answer any resulting research questions—the importance of which was demonstrated above.

The ultimate goal of this introductory paper is to foster such fruitful lines of conceptualizing research. The more proximal goal is to provide an accessible yet comprehensive exposition of a number of time series modeling techniques fit for addressing a wide range of research questions. These models were based in descriptive, explanatory, and predictive frameworks—all three of which are necessary to accommodate the complex, dynamic nature of psychological theory and its data.

### Conflict of interest statement

The authors declare that the research was conducted in the absence of any commercial or financial relationships that could be construed as a potential conflict of interest.

## Footnotes

^1^These journals were: *Psychological Review, Psychological Bulletin, Journal of Personality and Social Psychology, Journal of Abnormal Psychology, Cognition, American Psychologist, Journal of Applied Psychology, Psychological Science, Perspectives on Psychological Science, Current Directions in Psychological Science, Journal of Experimental Psychology: General, Cognitive Psychology, Trends in Cognitive Sciences, Personnel Psychology, and Frontiers in Psychology*.

^2^The specific search term was, “jobs – Steve Jobs” which excluded the popular search phrase “Steve Jobs” that would have otherwise unduly influenced the data.

^3^Thus, the highest value in the series must be set at 100—i.e., 100% of itself. Furthermore, although measuring a variable in terms of percentages can be misleading when assessing practical significance (e.g., a change from 1 to 4 yields a 400% increase, but may not be a large change in practice), the presumably large raw numbers of searches that include the term “jobs” entail that even a single point increase or decrease in the data is notable.

^4^In addition to the two classical models (additive and multiplicative) described above, there are further techniques for time series decomposition that lie beyond the scope of this introduction (e.g., *STL* or *X-12-ARIMA* decomposition). These overcome the known shortcomings of classical decomposition (e.g., the first and last several estimates of the trend component are not calculated; Hyndman and Athanasopoulos, 2014) which still remains the most commonly used method for time series decomposition. For information regarding these alternative methods the reader is directed to Cowpertwait and Metcalfe (2009, pp. 19–22) and Hyndman and Athanasopoulos (2014, chap. 6).

^5^Importantly, the current paper discusses dynamic models that specify time as the regressor (either as a linear or polynomial function). For modeling substantive predictors, more sophisticated techniques are necessary, and the reader is directed to Pankratz (1991) for a description of this method.

^6^Just like in traditional regression, the parent term *t* is centered before creating the polynomial term in order to mitigate collinearity.

^7^The use of additional fit indices, such as the AIC c (a variant of the AIC for small samples) and Bayesian information criterion (BIC) is also recommended, but we focus on the AIC here for simplicity.
