# Supplementary material for: Time series analysis for psychological research: examining and forecasting change
Source: Front Psychol. 2015 Jun 9;6:727. doi: 10.3389/fpsyg.2015.00727 (PMC4460302; doi:10.3389/fpsyg.2015.00727)
Supplement: Supplementary file 1 [file DataSheet1.PDF]

Appendix

R Tutorial for Implementing Time Series Analyses

Creating a Time Series Object

The function `ts` creates a time series object. The first argument is a data object that contains the observations of the time series variable. The `start` and `end` arguments specify the beginning and end times of the series and are specified as natural time units (here, years), with the following number (e.g., `(2004, 1)` indicating the particular point within that unit in which the series began and ended. The `frequency` argument denotes how many observations are contained in one time period (here, 12 months within one-year periods).

```
series = ts(data, start = c(2004, 1), end = c(2011, 6),
frequency = 12)
```

Time Series Decomposition

The `decompose` function partitions a time series into its seasonal, trend, and irregular components (Again, the duration of cyclical behavior is undefined and so cannot be extracted.) Additive or multiplicative decomposition can be specified using the `type` argument.

```
tsdecomp = decompose(series, type = ”additive”)
```

Seasonal Adjustment

The original series can be seasonally adjusted by performing the `seasadj` function in the forecast package (Hyndman, 2014) on a decomposed time series object.

```
tsadj = seasadj(tsdecomp)
```

Transformation to Stationarity

To stabilize the mean, an order of differencing can be applied to a series using the `diff` function, after which the variance should be reduced. The `adf.test` command in the tseries package (Trapletti & Hornik, 2013) implements an augmented Dickey-Fuller test for stationarity.

```
dseries = diff(series)
var(series)
adf.test(series)
```

If the mean of the series is not stationary, another round of differencing can be applied in the same way.

```
ddseries = diff(dseries)
var(ddseries)
adf.test(ddseries)
```

Non-stationary series variance can be addressed by working with the logarithm of the series.

```
logseries = log(series)
```

Plotting the Autocorrelation Function (ACF) and Partial Autocorrelation Function (PACF)

The functions `acf` and `pacf` produce plots of the autocorrelation and partial autocorrelation of a series, respectively.

```
acf(series)
pacf(series)
```

Modeling the Series as a Function of Time

A deterministic time series trend can be modeled as a linear or higher-order function of time. In either case, one needs to create a variable that represents time. For our example, there were 90 equally-spaced observations, so the `seq` command was used to generate a sequence from 1:90.

```
time = seq(from = 1, to = 90, by = 1)
```

If higher order terms are included, this parent term should be mean-centered to mitigate collinearity.

```
time = time - mean(time)
time2 = time^2
time3 = time^3
```

Finally, each regression model can be fit through the `gls` command which implements the generalized least squares estimator. Unlike ordinary least squares, this estimator can account for autocorrelated residuals which are often found in time series data. This function is available in the nmle package (Pinheiro, Bates, DebRoy, Sarkar, & R Core Team, 2014).

```
linear = gls(series ~ time)
quadratic = gls(series ~ time + time2)
cubic = gls(series ~ time + time2 + time3)
```

Interrupted Time Series Analysis

Again, one needs to first code a variable that represents time.

```
time = seq(1, 90, 1)
```

Then, a dummy variable must be created (here, `event`) that codes whether each observation occurred before the event (0) or after (1). In our data, there were 48 observations before and 42 after. This can be easily done using the `rep` function.

```
event = c(rep(0, times = 48), rep(1, times = 42))
```

One must then create a variable that codes how long after the event each observation occurred. (And pre-event observations are coded as zeros.)

```
tai = c(rep(0, times = 48), seq(from = 1, to = 42, by = 1))
```

One can then simply use these predictors in a regression model.

```
its = gls(tsadj ~ time + event + tai)
```

In our example, the post-event trend was modeled as a quadratic function, which can be accomplished by mean-centering the post-event dummy variable term (`tai`) and including a squared term (`tai2`).

```
tai = tai - mean(tai)
tai2 = tai^2
its = gls(tsadj ~ time + event + tai + tai2)
```

Estimating Seasonal Effects

Seasonal indicator models. Estimating seasonal effects requires building a regression model regressing on time with a constant for each season. As in the previous analyses, a dummy variable representing time is created, as well as a categorical seasonal variable. The latter is performed by the `cycle` function, which identifies the season of each observation.

```
time = seq(from = 1, to = 90, by = 1)
seas = cycle(series)
```

The `factor` function redefines the object as a factor.

```
seas = factor(seas)
```

Then, a regression model with seasonal indicators can be specified. Importantly, the first term in the model should be a zero so that no general intercept is estimated, otherwise a seasonal term will be left out.

```
model = gls(series ~ 0 + time + seas)
```

Harmonic seasonal models. A harmonic seasonal model is more complex than a model with an indicator per season, but will usually be more parameter efficient and provide a more realistic approximation. (This section was adapted from Cowpertwait & Metcalfe, 2009, pp. 103-104). First, a variable representing time should be created.

```
time = seq(from = 1, to = 90, by = 1)
```

Then, predictors with either a sine or cosine term should be created that represent the seasonal pattern. The formula for this values comes from Equation 9, where, for *S* seasons, there are *S*/2 *s* and *c* coefficients (here, six of each).

```
sin1 = sin(2 * pi * 1 * time/12)
cos1 = cos(2 * pi * 1 * time/12)
sin2 = sin(2 * pi * 2 * time/12)
cos2 = cos(2 * pi * 2 * time/12)
…
sin6 = sin(2 * pi * 6 * time/12)
cos6 = cos(2 * pi * 6 * time/12)
```

The series is then regressed on all of these terms.

```
model = gls(ts ~ time + sin1 + cos1 + sin2 + cos2 + sin3 +
cos3 + sin4 + cos4 + sin5 + cos5 + sin6 + cos6)
```

The statistically significant coefficients can be identified through the `summary` function.

```
summary(model)
```

Only the statistically significant coefficients in the model should be retained. For our data, only five were kept, and the model was re-specified.

```
model = gls(ts ~ time + cos1 + sin2 + cos3 + sin3 + cos5)
```

Dynamic Regression Modeling (ARIMAX)

The `Arima` function found in the forecast package fits an ARIMA model to the data. The `order` argument specifies the orders of the ARIMA model (in our example, one AR term and one order of differencing), while the argument `xreg` defines which data object contains the observations of the predictors. To fit an ARIMA model without external predictors, simply omit the `xreg` argument.

```
model = Arima(series, order = c(1,1,0), xreg = covariates)
```

Additionally, the `auto.arima` function also found in the forecast package automatically locates the best-fitting ARIMA model to the data, “fit” defined by one of three possible information criteria in the `ic` argument: the AIC (given by `“aic”`), the AIC_c (`“aicc”`), or the BIC (`“bic”`).

```
model = auto.arima(series, ic = “aic”)
```

Residual Analysis

The `residuals` function extracts the residuals from a fitted model object.

```
res = residuals(model)
```

One can formally identify any remaining autocorrelation from a regression model by a Durbin-Watson test (`dwtest`) in the lmtest package (Zeileis & Hothorn, 2002).

```
dwtest(model)
```

The function `Box.test`, with the type argument set as `“Lj”`, conducts a Ljung-Box test on the residuals after an ARIMA model has been fit.

```
Box.test(res, type = "Lj")
```

Forecasting

The `forecast` function in the forecast package can be used to generate predictions from a model, with the `h` argument specifying how many periods ahead to forecast. If the forecasting model includes external predictors (i.e., an ARIMAX model), then projected or known values of these covariates for the `h` periods ahead must be provided in the `xreg` argument. If there are no external predictors (i.e., an ARIMA model), then this argument can simply be dropped.

```
fcasts = forecast(model, xreg = covariates, h = 6)
```

To assess the accuracy of a forecasting model, the `accuracy` function, also found in the forecast package, produces accuracy statistics, such as the MAE and MAPE. The `x` argument to this function provides the actually observed values (here, contained in an object titled `observed`) with which to compare the produced forecasts.

```
accuracy(fcasts, x = observed)
```
